# Structural basis of Mlc-mediated transcriptional regulation of carbohydrate metabolism

**DOI:** 10.1038/s41467-026-75270-8

**Published:** 2026-07-11

**Authors:** Patrick Roth, Inken Fender, Jean-Marc Jeckelmann, Zöhre Ucurum, Thomas Lemmin, Dimitrios Fotiadis

**Affiliations:** Institute of Biochemistry and Molecular Medicine, Medical Faculty, University of Bern, Bern, Switzerland

**Keywords:** Cryoelectron microscopy, Cryoelectron microscopy, Transcriptional regulatory elements

## Abstract

The global transcriptional repressor Mlc of *Escherichia coli* regulates genes involved in carbohydrate transport and metabolism, particularly glucose uptake via the glucose-specific phosphotransferase system (PTS). Unlike conventional repressors, Mlc exemplifies a system in which interactions with diverse macromolecules govern its activity. Here, we present cryo-electron microscopy structures of Mlc alone and in complexes with regulatory partners, including the glucose-specific PTS transporter IICB^Glc^, a cognate DNA operator and the anti-repressor MtfA, capturing multiple assemblies central to transcription control. These structures reveal the molecular architecture of Mlc and its interactions with binding partners. Together with molecular dynamics simulations, they provide insights into the structural dynamics of these complexes. Our findings establish the structural basis of membrane-transporter involvement in transcriptional regulation, the mechanism of anti-repressor action and DNA recognition. This work provides a structural framework for understanding bacterial transcriptional regulation across diverse systems.

## Abstract (web summary)

Bacterial repressors regulate transcription in response to molecular and environmental signals. Here, the authors show how Mlc binds a membrane glucose transporter, DNA and an anti-repressor, revealing steric control of transcription by distinct binding partners.

## Introduction

Bacterial persistence under fluctuating nutrient supply is mediated by tightly regulated gene expression networks that allow rapid adaptation to environmental changes. Carbohydrates are a major source of carbon and energy, fueling metabolism and serving as biosynthetic precursors, making their acquisition and utilization essential for bacterial growth and survival. Insights into carbohydrate metabolism and regulation are fundamental for understanding microbial survival, resistance and pathogenesis, for optimizing biotechnological processes and for developing novel therapeutic strategies^1–3^. For many bacteria, glucose (Glc) is the most important and preferred carbohydrate, and is preferentially acquired through the hierarchically regulated carbon catabolite repression process^4^. The widespread bacterial phosphoenolpyruvate (PEP)-dependent phosphotransferase system (PTS) is a multi-component transport system that integrates carbohydrate sensing and uptake with metabolic regulation^5–8^, particularly through modulation of transcription factor activity^9^. These transcription factors regulate gene expression by binding operator regions and modulating RNA polymerase access to promoters^10,11^. The glucose-specific PTS of *Escherichia coli* couples glucose uptake with cytoplasmic phosphorylation through the sequential transfer of a phosphoryl group from PEP to enzyme I (EI), the histidine-containing phosphocarrier protein (HPr), the soluble IIA^Glc^ and finally the IIB^Glc^ domain of the membrane-associated IICB^Glc^ transporter, which transfers the phosphoryl group to glucose bound to IIC^Glc^, yielding glucose-6-phosphate^12^. The transcriptional repressor Mlc (*making large colonies*)^13^ is a global regulator of carbohydrate metabolism, controlling genes involved in carbohydrate utilization, particularly those related to glucose metabolism^14,15^. Unlike numerous repressors that respond to small-molecule effectors through allosteric conformational changes^16^, Mlc activity is controlled by protein-protein interactions^17^, a regulatory principle still poorly understood at the molecular level. For Mlc repressed genes, two induction mechanisms have been proposed. First, repression is relieved by the sequestration of Mlc away from DNA, through its binding to the dephosphorylated IIB^Glc^ domain of the IICB^Glc^ transporter located at the plasma membrane^18–24^. The second induction mechanism involves the soluble Mlc titration factor A (MtfA), which acts as a putative anti-repressor on Mlc, also sequestering Mlc away from DNA^25–27^. Notably, MtfA features a metalloprotease fold, but its substrate specificity is unknown^26^.

Mlc belongs to the functionally diverse ROK (Repressors, Open reading frames and Kinases) family and characteristically contains a conserved cysteine-rich motif involved in zinc cation coordination. Each Mlc protomer comprises three domains responsible for oligomerization, regulatory protein binding and DNA-binding via a conserved helix-turn-helix (HTH) motif^24,28^. As a pleiotropic repressor, Mlc in *E. coli* regulates multiple genes and operons, including *ptsG* (encoding IICB^Glc^), *ptsHIcrr* (encoding the cytoplasmic PTS components HPr, EI and IIA^Glc^), *manXYZ* (encoding the mannose-specific PTS components IIAB^Man^, IIC^Man^ and IID^Man^), *malT* (encoding the MalT transcriptional activator of the maltose regulon) and *mlc* itself and recognizes pseudo-palindromic operator sequences in the respective promoter regions^17,29^. Despite the central role of Mlc in regulating bacterial carbohydrate utilization and contributing to long-term survival in *E. coli*^30^, several aspects remain unresolved, including its oligomerization state, the architecture and molecular basis of interactor recognition and binding, and the associated dynamics.

To address these open questions, we employed a structural approach based on cryo-electron microscopy (cryo-EM) and molecular dynamics (MD) simulations. We report four cryo-EM structures of Mlc from *E. coli*, i.e., in isolation, bound to a cognate DNA operator, and in complexes with the PTS glucose transporter IICB^Glc^ and the anti-repressor MtfA, capturing key complexes of this transcriptional regulatory circuit. These structures reveal the supramolecular organization of the complexes and provide the molecular basis for Mlc interactions with regulatory binding partners, and, together with molecular dynamics simulations, uncover dynamic properties of these assemblies.

## Results

### Structure of the apo-Mlc oligomer

**Fig. 1:**
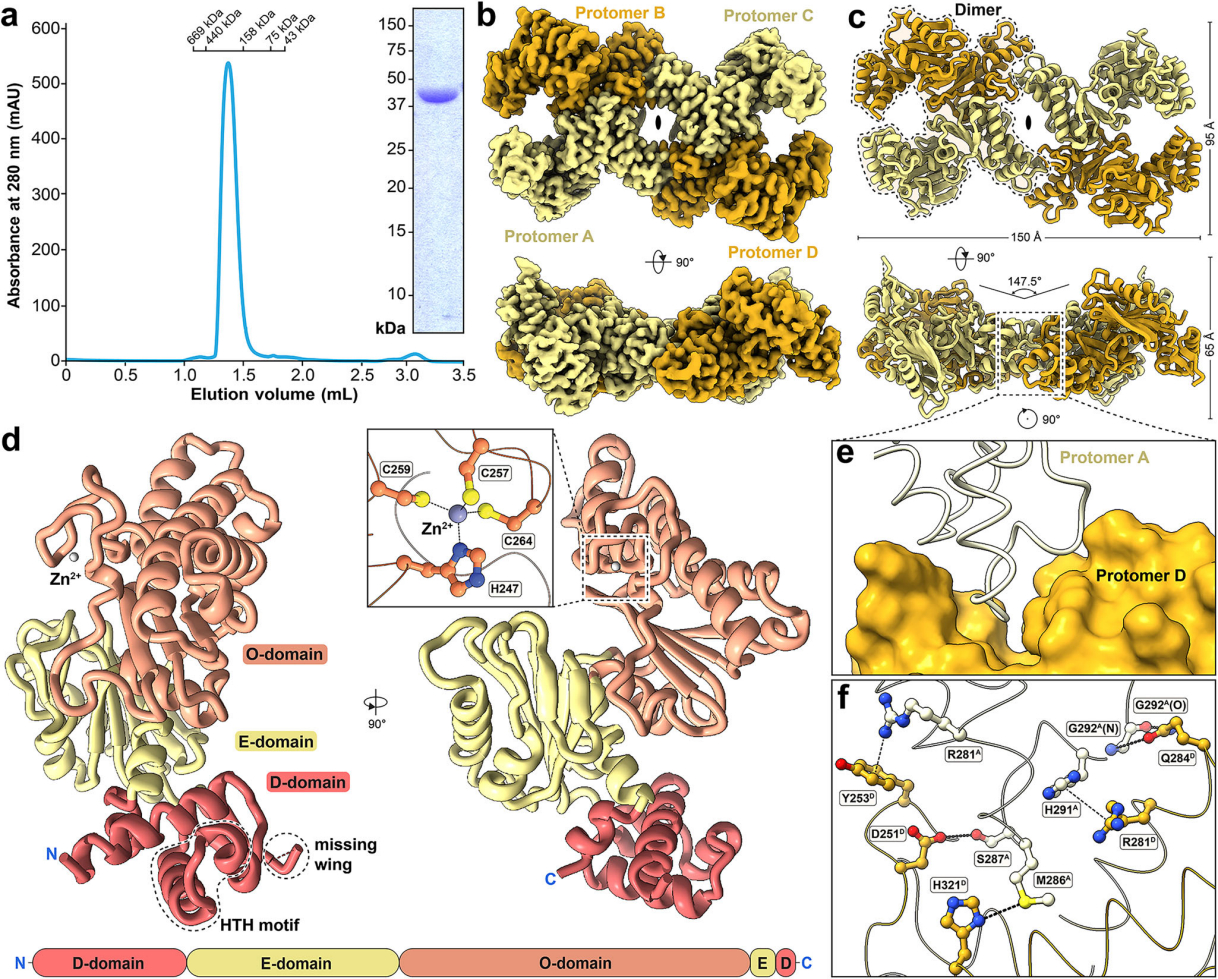
Biochemical characterization and structure of tetrameric apo-Mlc. **a** Size-exclusion chromatography (SEC) profile and Coomassie Brilliant Blue-stained SDS-PAGE gel of Mlc used for structural analysis. Elution volumes of molecular-weight standards are indicated above the elution peak. SEC experiments were repeated more than three times with comparable results. Source data are provided as a Source Data file. mAU, milli-absorbance units. **b** Cryo-EM density map of the tetrameric apo-Mlc complex shown from two orthogonal views. Symmetry-related protomers (i.e., A/C and B/D) are colored in yellow and orange. **c** Corresponding structural model with overall complex dimensions indicated. One dimer of the tetrameric dimer-of-dimers assembly is contoured. The C_2_ symmetry axis (perpendicular to the viewing plane) is marked with a filled ellipse in (**b**) and (**c**). **d** Structure of the Mlc protomer and its domain organization. The D-, E- and O-domains are shown in distinct colors, with key structural elements highlighted, e.g., the Zn^2+^-binding site and the helix-turn-helix (HTH) motif. The letters N and C, shown in blue, indicate the amino- and carboxy-termini, respectively. The inset displays the tetrahedral coordination of the Zn^2+^ ion by the C3H1-type zinc finger motif, with interaction distances of 2.3 Å between Zn^2+^ and the cysteine sulfur atoms and 2.1 Å between Zn^2+^ and the proximal histidine nitrogen atom. The linear representation (*bottom*) illustrates the domain organization of Mlc. **e** View of the tetramer interface mediating formation of the dimer-of-dimers complex, as shown in (**c**), but rotated by 90° in-plane and slabbed. The O-domain of protomer D is shown as an orange surface, while the O-domain of the interacting protomer A is depicted as a pale-yellow cartoon occupying the cleft of protomer D. **f** Molecular interactions (distances ≤ 4 Å) at the tetramerization interface, viewed as in (**e**). N and O in brackets refer to backbone amine and carbonyl interactions, respectively, and superscript letters denote the protomers.

Mlc from *E. coli* was homologously overexpressed and purified by nickel affinity chromatography, followed by proteolytic removal of the His-tag. Subsequent size-exclusion chromatography (SEC) revealed a homogenous species with an apparent molecular weight of ~ 200 kDa, consistent with a putative homotetramer composed of ~ 44 kDa protomers (Fig. 1a). Single-particle cryo-EM was employed to determine the apo-Mlc oligomer structure, and obtained reconstructions at global resolutions of 2.6–2.9 Å (Supplementary Fig. 1) were merged into a composite map (Supplementary Fig. 2a). The cryo-EM density map of apo-Mlc revealed a homo-tetrameric, dimer-of-dimers assembly with a central hole and overall C_2_-symmetry (Fig. 1b, c). The double X-shaped complex has dimensions of 150 × 95 × 65 Å and appears bent by 147.5° when viewed from the side (Fig. 1c). The protomers exhibit characteristic fold features of the ROK family repressors, and comprise three functional domains, which are the D- (DNA-binding domain, residues 1–81 and 396–406), E- (IIB^Glc^-binding domain, residues 82–194 and 380–395) and O-domain (oligomerization domain, residues 195–379). Structural C-terminal backfolding contributes to the D- and E-domains (Fig. 1d). The α-helical D-domain (α1–3 and α12; Supplementary Fig. 3a) features a helix-turn-helix (HTH) motif and wing with conserved amino acid residues (Supplementary Fig. 4a). The N-terminal part of the E-domain comprises seven β-sheets interrupted by two short α-helical stretches, forming a scaffold for the C-terminal part of the E-domain, which then transitions into the C-terminal part of the D-domain (Fig. 1d and Supplementary Fig. 3a). The residues constituting the N-terminus and the wing following the HTH motif were not resolved, probably due to inherent flexibility or conformational heterogeneity (Supplementary Fig. 1g). The large O-domain contributes to oligomerization and consists of a continuous polypeptide, composed of five relatively long α-helices and four β-strands (Supplementary Fig. 3a). As typical for ROK family proteins, Mlc comprises the conserved Zn^2+^-binding motif in the O-domain. H247 and three cysteines (C257, C259 and C264) form a C3H1-type zinc finger with tetrahedral coordination of the metal cation (Fig. 1d).

The oligomeric state of Mlc arises from two different interfaces (Supplementary Fig. 3b): The two protomers forming the dimer interact by an intertwinement via the corresponding faces of their O-domains (dimerization contacts). This interface buries a surface area of approximately 1373 Å^2^ and features a central hydrophobic patch surrounded by polar residues contributing with several hydrogen bonds to dimerization (Supplementary Fig. 3c). The zinc finger motifs of the two protomers are near the dimerization interface (Supplementary Fig. 3c). The symmetric dimer-of-dimers assembly is formed between the O-domains of two dimerized Mlc protomers. The tetramerization interface is formed by tip-to-groove interactions between opposite O-domains (Fig. 1e). These interactions result in a relative rotation by 17° between the two dimers constituting the tetramer (Supplementary Fig. 3b). The mentioned tip-to-groove tetramerization contacts are primarily formed by an ordered loop between α7 and α8 forming the tip on one side, while the groove on the opposite protomer is shaped by α7 and α9, and a loop structured by the zinc finger motif. The tetramerization surface buries an area of approximatively 1130 Å^2^ with considerable electrostatic contributions: Two hydrogen bonds between S287^A^/D251^D^ (superscript letters denote Mlc protomers) and G292^A^ amino and carbonyl backbone with the side chain of Q284^D^ as well as cation-π and π-stacking interactions of arginine residues R281^A^ and R281^D^ with Y253^D^ and H291^A^, respectively, are involved (Fig. 1f). The protruding side chain of M286^A^ inserts deep into the cleft of the opposite protomer and forms an interaction with the H321^D^ side chain (Fig. 1f).

### Structure of the Mlc:DNA complex

**Fig. 2:**
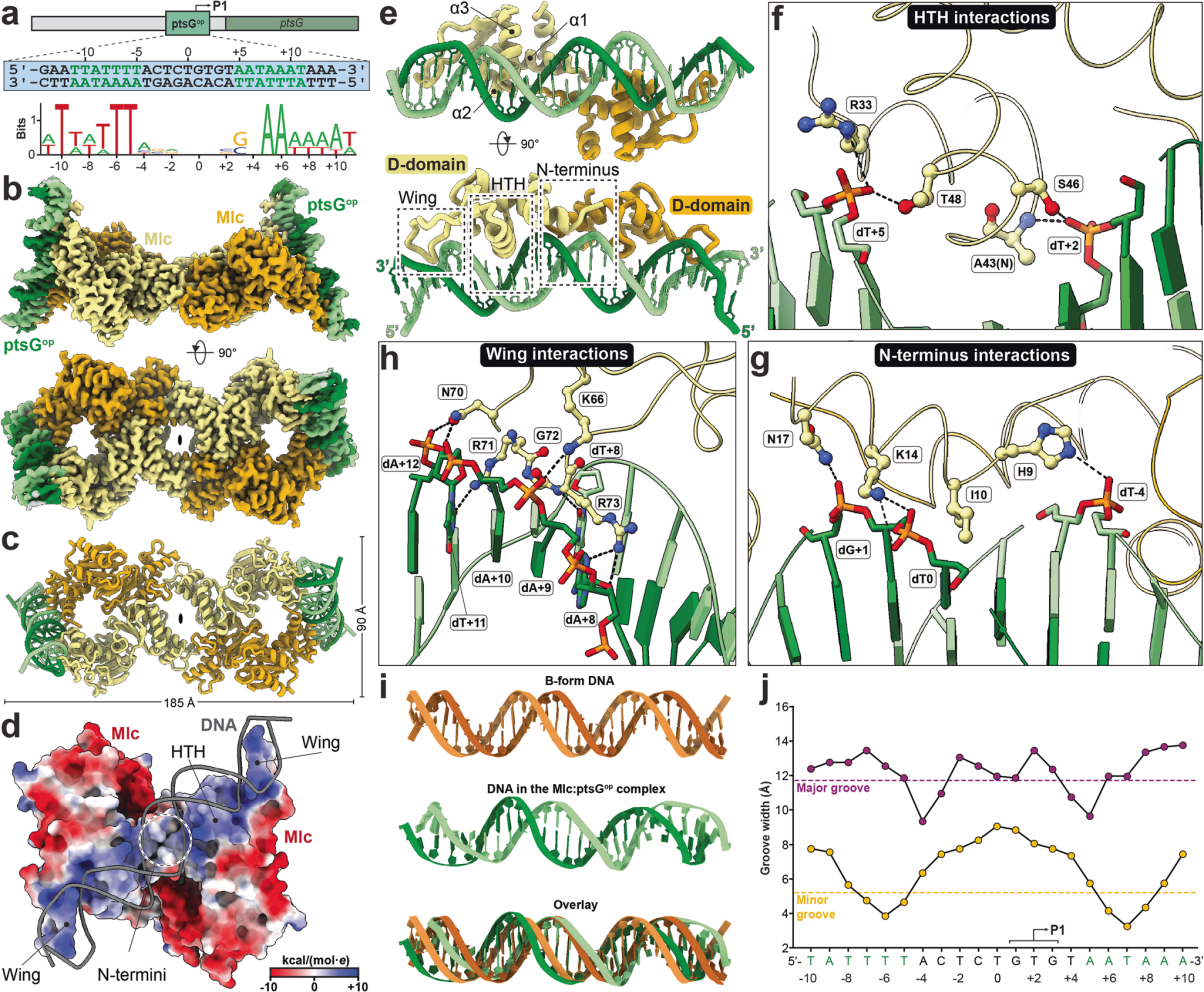
Structure of Mlc in complex with DNA (Mlc:DNA). **a** Organization of the *ptsG* gene (encoding the IICB^Glc^ transporter) promotor region, showing the Mlc operator binding site (box; ptsG^op^) and the transcription start site P1 (GTG start codon at + 1). The DNA sequence of the ptsG^op^ site is shown enlarged. DNA sequence conservation analysis (sequence logo) of known Mlc binding sites is displayed below, with positions conventionally numbered relative to the center (set to zero); the left and right half-operators are assigned negative and positive position values, respectively. The conserved pseudo-palindromic A/T-rich sequences flanking the non-conserved central region are evident. Source data are provided as a Source Data file. **b** Composite cryo-EM density map of the Mlc:DNA complex. Mlc protomers are colored yellow and orange, and the operator DNA is in green tones. **c** Structural model and dimensions of the Mlc:DNA complex. The C_2_ symmetry axis (perpendicular to the viewing plane) is marked with a filled ellipse in (**b**) and (**c**). **d** View on Mlc rendered by the calculated Coulomb electrostatic potential with bound DNA (gray cartoon), revealing a basic surface aligned with the DNA. Red and blue indicate negative and positive surface charge potentials (color bar in kcal/(mol·*e*)). **e** Cartoon representation of the Mlc-DNA interface. Three protein-DNA interaction regions were identified: the wing, the helix-turn-helix (HTH) motif and the N-terminal region, with the corresponding molecular contacts (distances < 4 Å) shown in panels (**f**–**h**). Hydrogen bond and salt bridge interactions are shown as dashed lines. **i** Comparison of the DNA structure in the complex with Mlc and regular B-form DNA reveals a bending. **j** Minor (orange) and major (magenta) groove widths plotted along the length of the resolved Mlc-bound DNA. Values represent the distances between the closest interstrand backbone phosphates after subtracting for van der Waals radii. Dashed lines indicate the corresponding groove widths of ideal B-form DNA. The transcription start site P1 is indicated.

We selected the DNA sequence of the Mlc binding site within the *ptsG* promotor (operator region) for structural experiments, due to its high affinity of 1.75 nM^3^ to Mlc. This operator, hereinafter referred to as ptsG^op^, specifically co-regulates the expression of the glucose-specific PTS transporter IICB^Glc^ and comprises the transcription start point P1 (Fig. 2a). The complex was assembled from Mlc and the double-stranded (ds) ptsG^op^ DNA, yielding a stable complex as evidenced by co-elution in analytical SEC (Supplementary Fig. 5a). We then determined the Mlc:DNA complex structure by cryo-EM (Supplementary Fig. 5b–g). The obtained composite map featured excellent densities for both the protein and the oligonucleotide (Fig. 2b and Supplementary Fig. 2b), enabling the construction of a detailed atomic model of the repressor-operator complex. The density for the D-domains was better defined than in the apo structure, and additional residues for the N-terminal region and the previously unresolved wing (residues 63–76) could be modeled. The complex has dimensions of 185 × 90 × 90 Å. Similar to the apo structure, Mlc adopts a dimer-of-dimers, homo-tetrameric assembly, and each dimer binds one double-stranded operator, yielding a hetero-hexameric complex with Mlc_4_:dsDNA_2_ stoichiometry (Fig. 2c). The central axis of the DNA aligns with a continuous, positively charged surface provided by the dimer’s D-domains, which stabilizes the negatively charged phosphate backbone (Fig. 2d). Thereby, a near-symmetric interface with the pseudo-palindromic operator is formed. The two D-domains of an Mlc dimer simultaneously engage both halves of the operator sequence, sterically occluding the P1 start codon and efficiently blocking access to the RNA polymerase. Both D-domains establish numerous interactions with both DNA strands. The binding interface between an Mlc protomer and the half-operator can be divided into three regions: An N-terminal contact region, an HTH-motif-interacting region and a wing-interacting region (Fig. 2e). As in other HTH-containing repressors, the HTH motif in the Mlc D-domain inserts into the half-operators’ major groove, mediated by the C-terminal end of the recognition helix (α3, Fig. 2e). Surprisingly, no canonical base-specific contacts of the recognition helix were observed; instead, only interactions with the phosphate backbone were detected, raising the question of how sequence specificity is achieved. These DNA sequence-unspecific contacts involve the Mlc side chains of S46 and T48 as well as the backbone nitrogen of A43, all within hydrogen-bonding distance to backbone phosphate groups (Fig. 2f). The adjacent positioning helix (α2) of the HTH adopts a V-like shaped arrangement relative to the recognition helix and is oriented approximately perpendicular to the major groove. It also forms an additional salt bridge between R33 and the phosphate group of dT+5 (Fig. 2f). The N-terminal helices (α1) of both protomers interact with the center of the operator (around the zero position) in a symmetrical manner (Fig. 2e). These interactions are with amino acid side chains and backbone phosphates, i.e., H9/dT-4, K14/dT0 and N17/dG+1 (Fig. 2g). The tips of these α1-helices partially insert into the widened minor groove via the I10 residue. Another interaction between Mlc and the operator is formed by the Mlc’ peripheral wings (Fig. 2e). Unlike in apo-Mlc, this newly resolved stretch (wing) undergoes a disorder-to-order transition upon operator binding, inserting as a loop into the peripheral minor grooves of the operator. The tips of the wings, formed by the amino acid sequence R71-G72-R73, insert into the minor grooves centered at positions ± 9 (relative to G72). Within these tips, the side chains of R71 and R73 adopt opposite orientations and align parallel to the floor of the minor groove, while G72 does not directly participate in contacts (Fig. 2h). The positively charged guanidinium groups of the two arginine residues penetrate deeply into the anionic minor groove (Supplementary Fig. 4b), attracted by the highly negative electrostatic potential at these A/T-rich sites, which represent a conserved hallmark of cognate Mlc operator sequences (Fig. 2a). Specifically, the side chain of R71 interacts with the base of dT + 11, while the side chain and backbone amine of R73 interact with the nucleotides dA+8 and dT+8. Further wing contacts include a salt bridge between K66 and the phosphate of dA + 10, and hydrogen bonds formed between N70 and the backbone of dT + 11 and dA + 12 (Fig. 2h). The overall conformation of the DNA operator is substantially altered upon Mlc binding compared with ideal B-form DNA (Fig. 2i). Specifically, both left and right half-operators bend upon interaction with the D-domains, inducing distinct local deformations. The average roll and twist angles of the Mlc-bound DNA are 1° and 29° compared with 0° and 36° in ideal B-form DNA. Interactions involving the wing domains and N-terminal regions of Mlc widen the protein-facing minor groove openings while simultaneously narrowing the major grooves (Fig. 2j). The interactions of Mlc with the other half-operator of the pseudo-palindromic stretch are essentially the same.

### Structure of the Mlc:IICB^Glc^ transporter complex

**Fig. 3:**
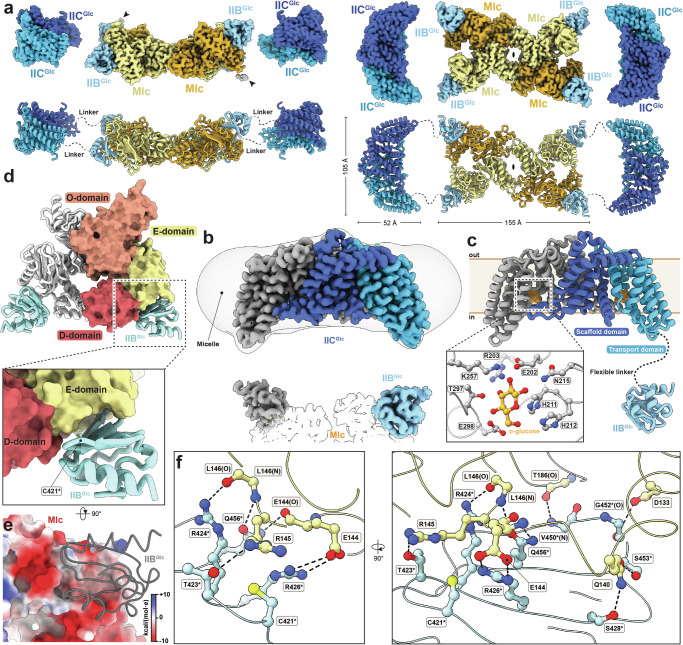
Structure of Mlc in complex with the glucose transporter IICB^Glc^ (Mlc:IICB^Glc^). **a** Composite cryo-EM density map of the hetero-octameric Mlc:IICB^Glc^ complex (*top*) and the corresponding structural models (*bottom*) with a 4:4 stoichiometry. Mlc protomers are shown in yellow and orange, whereas IICB^Glc^ is depicted in blue tones, with the IIB^Glc^ domain that interacts with Mlc highlighted in sky blue. Dimensions of the complex are indicated, and dashed lines denote the unresolved IIC^Glc^-IIB^Glc^ linker. The C_2_ symmetry axis (perpendicular to the viewing plane) is marked with a filled ellipse (*right*). Densities corresponding to the disordered wing regions are shown in gray and indicated with arrowheads. **b** Front view of the cryo-EM density map of dimeric IICB^Glc^ in complex with Mlc (in white). One IICB^Glc^ protomer is highlighted gray, and the other in blue shades, and the detergent micelle is rendered as a transparent gray surface. **c** Model of the IICB^Glc^ transporter embedded in the plasma membrane, illustrating the functional scaffold and transport IIC^Glc^ domains together with the flexibly linked IIB^Glc^ domain (one shown, colored as in the previous panel). Both protomers adopt a glucose-bound, inward-facing, occluded conformation. The inset depicts the specific coordination of D-glucose (orange) by binding site residues of IIC^Glc^. **d** Overview of the domain arrangement in the Mlc:IIB^Glc^ interaction (*top*) and zoom-in view (*bottom*). Oligomerization- (O, orange), DNA- (D, red) and IIB^Glc^-binding (E, yellow) domains of Mlc and the IIB^Glc^ (sky blue) protein are labeled. The location of the dephosphorylated, active-site cysteine residue (C421) in IIB^Glc^ is highlighted. **e** Mlc surface (related to panel (d)) colored by Coulomb electrostatic potential (color bar in kcal/(mol·*e*)), with IIB^Glc^ shown as gray ribbon. **f** Orthogonal views of the interactions between residues of Mlc E-domain (yellow) and IIB^Glc^ (sky blue). Dashed lines indicate hydrogen bonds and a salt bridge (distances ≤ 3.5 Å), and the location of C421 is shown. An asterisk sign denotes residues in IICB^Glc^.

To obtain structural insights into the membrane-mediated sequestration of Mlc, we assembled the complex from separately purified Mlc and glucose-specific PTS transporter IICB^Glc^. SEC analysis showed a distinct shift relative to the individually run constituents, yielding a defined high-molecular weight species (Supplementary Fig. 6a). Cryo-EM analysis revealed elongated particles (Supplementary Fig. 6b). However, the intrinsic flexibility of the complex hindered high-resolution structure determination. A consensus 3D reconstruction using a soft mask enclosing the entire complex indicated pronounced flexibility in the peripheral regions, which appeared as blurred density features. 3D variability analysis confirmed our observations and revealed a continuous rocking and scissoring motion of both peripheries of ~ 45° relative to the central density (Supplementary Fig. 6c). Due to this flexibility, the central and peripheral parts were locally extracted and refined separately. This strategy yielded clean two-dimensional (2D) class averages for each part, while the corresponding adjacent part appeared substantially blurred (Supplementary Fig. 6d, e). Particle subsets were then successfully used for 3D reconstructions, reaching overall resolutions of 2.78–2.95 Å (Supplementary Fig. 6f–i). Using these focused maps (Supplementary Fig. 2c), a composite map of the entire complex was generated by fitting the locally processed maps in the consensus reconstruction (Fig. 3a). Since the placement of the peripheral regions is based on density maxima, the conformation shown in Fig. 3a likely represents only one of several possible arrangements. The complex is hetero-octameric, with a 1:1 stoichiometry of Mlc and IICB^Glc^ (Mlc_4_:IICB^Glc^_4_), amounting to a total molecular weight of ~385 kDa for all protein constituents. Mlc at the center of the complex assembles into a double X-shaped homo-tetramer. At the outer corners of each Mlc monomer, an IIB^Glc^ domain binds tightly. IIB^Glc^, the soluble, catalytic domain of the IICB^Glc^ transporter, is covalently linked via an unresolved flexible linker to the IIC^Glc^ membrane protein domain. The latter has a crescent shape when viewed from the membrane plane and comprises of two tightly interacting protomers forming a dimer (Fig. 3b, c). Each protomer consists of 10 transmembrane helices arranged into a scaffold and a transport domain. IIC^Glc^ was captured in an inward-facing conformation, as indicated by the cytoplasmically inclined transport domains. Overall, its architecture is similar to the previous structure solved in lipid nanodiscs^31^ (Cα r.m.s.d. of 0.6 Å between the two structures). A glucose molecule was identified in each substrate-binding site (Fig. 3c), interacting with IIC^Glc^ in the same manner as previously described^31^. In summary, the structure of the IIC^Glc^ transporter was solved in the inward-facing, occluded glucose bound state. The structure of the IIB^Glc^ protein was solved in complex with Mlc. The IIB^Glc^ structure adopts a split α/β-sandwich fold, consisting of two antiparallel β-sheets (β1/β2 and β3/β4) interrupted by a short α-helix (α2), and the N- and C-terminal α-helices α1 and α3 (Supplementary Fig. 4c). The IIB^Glc^ domain contains a hydrophobic core and a polar surface, including the active site C421*, which is transiently phosphorylated during glucose transport, at the edge of β1/β2 surrounded by R424* and R426* (asterisks denote residues in IIB^Glc^). Compared to previously published structures of IIB^Glc^, the low Cα r.m.s.d. values suggest no significant structural differences (Supplementary Fig. 4c). The complex interface between Mlc and IIB^Glc^ is mainly formed by a structured loop between α4-β4, as well as by α4 and α5 within the E-domain of Mlc; and the antiparallel β-sheets (β2, β3 and β4) of IIB^Glc^ (Fig. 3d and Supplementary Fig. 4c). Numerous hydrophobic and polar interactions ensure a tight packing between the IIB^Glc^ domain and Mlc protein, giving rise to a total interaction surface area of ~ 623 Å^2^ (Fig. 3e). One contact region involves polar interactions between the T186 carbonyl oxygen and the V450* amide nitrogen, and moreover the polar backbone atoms of L146 with R424* and Q456* (Fig. 3f). Another contact region comprises interactions between Q140 and S428*, and notably, a salt bridge between E144 and R426* (Fig. 3f). C421* is in a dephosphorylated state and is not involved in the complex interface (Fig. 3f).

### Structure of the Mlc:MtfA anti-repressor complex

**Fig. 4:**
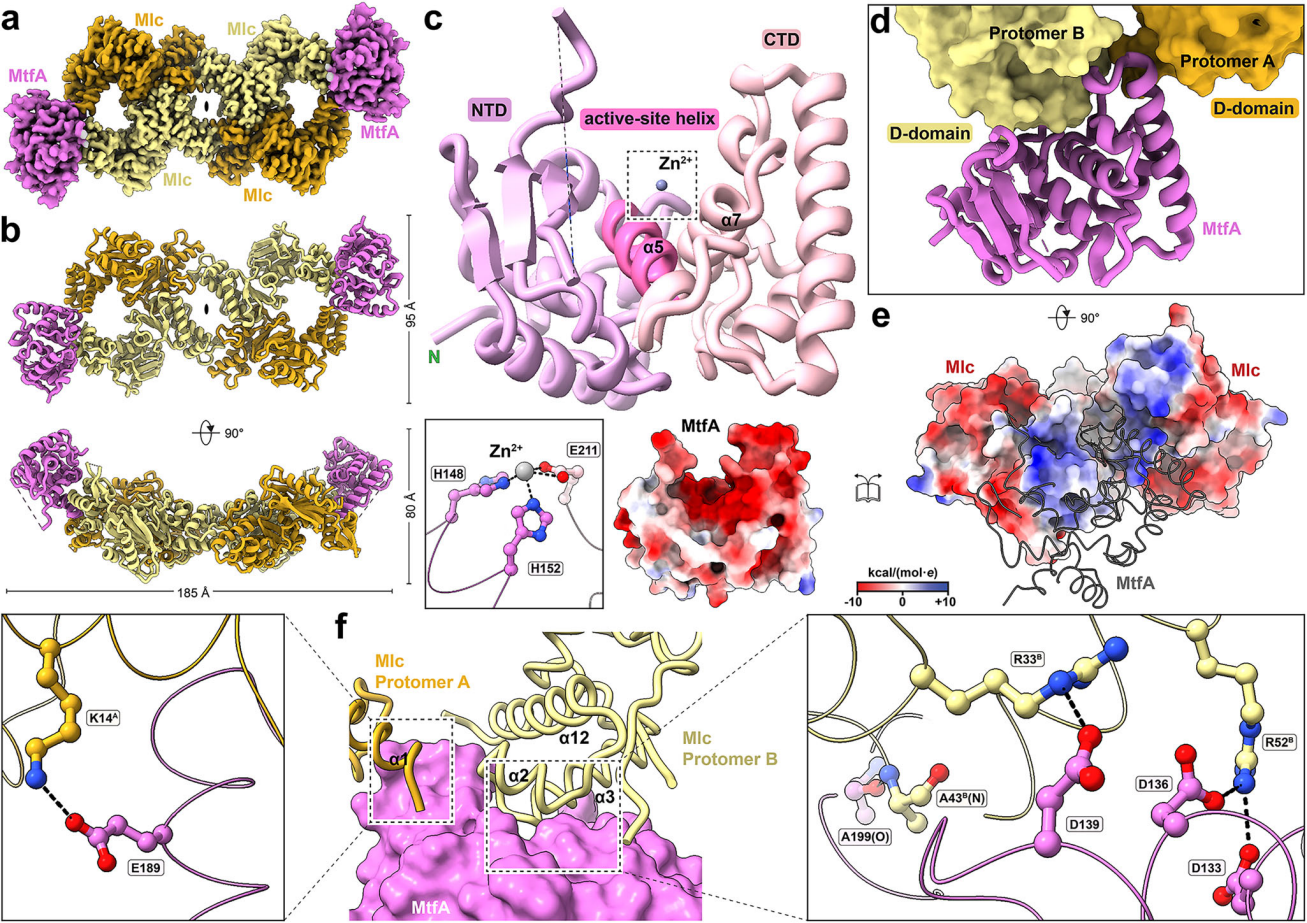
Structure of Mlc in complex with the anti-repressor MtfA (Mlc:MtfA). **a** Composite cryo-EM density map of the hetero-hexameric Mlc:MtfA complex with a 4:2 stoichiometry. Mlc protomers are shown in yellow and orange, and MtfA monomers in pink. **b** Structural model of the Mlc:MtfA complex with overall dimensions indicated, shown from two orthogonal perspectives. The C_2_ symmetry axis (perpendicular to the viewing plane) is marked with a filled ellipse in (**a**) and (**b**). **c** Structure of the MtfA monomer and its domain organization. The N-terminal (NTD) and C-terminal (CTD) domains are linked by a connecting helix (α5), and the amino terminus is indicated by N (green). A missing polypeptide stretch is indicated by a dashed line. The resulting cleft harbors the putative protease active site, featuring a Zn^2+^ ion coordinated by histidine and glutamate residues (*inset*). The surface representation of MtfA, colored by the calculated Coulomb electrostatic potential, reveals a negatively charged surface region surrounding the catalytic cleft; Red and blue indicate negative and positive surface charge potentials, respectively (color bar is in kcal/(mol·*e*)). **d** View of the interactions between the DNA-binding domains (D-domain, surface) of Mlc dimer and MtfA (cartoon model). **e** The surface of Mlc colored by the calculated Coulomb electrostatic potential, shows a complementary positively charged patch at the MtfA interaction site (MtfA is shown as a gray ribbon). **f** Molecular interactions at the Mlc:MtfA interface reveal hydrogen bonds and salt bridges (dashed lines, with distances ≤ 3.6 Å) contributed by both Mlc D-domains and MtfA. The overview in the center is rotated by 180° relative to the view in (**d**). N and O in brackets denote amine and carbonyl backbone interactions, and superscript letters A and B refer to the corresponding Mlc protomers.

To elucidate the structural basis for the MtfA-mediated de-repression mechanism of Mlc, we assembled the Mlc:MtfA complex using tetrameric Mlc and monomeric MtfA and determined its structure using cryo-EM at overall resolutions of 2.30 and 2.94 Å for focused maps (Fig. 4a and Supplementary Figs. 2, 7). The complex reveals a crescent-shaped architecture from the side and has dimensions of 185 × 95 × 80 Å (Fig. 4b). As in previous structures, Mlc forms the center of the complex. Each Mlc dimer binds one MtfA protein, yielding an Mlc_4_:MtfA_2_ stoichiometry and thus a hetero-hexameric assembly (Fig. 4a, b). Residues 16–100 and 124–251 of MtfA were resolved in the obtained 3D density map, while both termini and an internal loop appear flexible or disordered. The structure of MtfA comprises an N-terminal domain (NTD, residues 16–144) and a C-terminal domain (CTD, residues 159–253), connected by helix α5 (Fig. 4c). The NTD consists topologically of four α-helices (α1-4), three parallel β-sheet (β1-3) structures, while the CTD consists of three α-helices (α6-8) (Supplementary Fig. 4d). MtfA has been proposed as a zinc metallopeptidase; however, its physiological substrate remains unknown^26^. The putative active site forms a groove-shaped invagination formed by the NTD and CTD on each side and includes α5 (Fig. 4c). The two conserved zinc-binding consensus motifs ^148^HEXXH^152^ in helix α5 together with ^211^EXXA^214^ in α7 classifies MtfA as a metallopeptidase of the gluzincin clan^32^. The two histidine residues (H148 and H152) and the downstream glutamic acid residue (E211) coordinate a zinc ion (Fig. 4c). Overall, our MtfA structure from *E. coli* within the Mlc:MtfA complex aligns well with the reported active state MtfA structure from *Klebsiella pneumoniae* (Cα r.m.s.d. = 1.1 Å). Interestingly, MtfA interacts as a monomer with a dimer of Mlc, involving both D-domains in a wedging interaction with complementary charge and shape (Fig. 4d, e). The intermolecular interactions between the Mlc dimer and MtfA occur at two spatially different sites: The first contact site is formed with the D-domain of one Mlc protomer and buries an area of ~292 Å^2^. Here, the N-terminal α1 helix of Mlc is stabilized by a salt bridge between K14^A^ and E189 (superscript letters denote the protomers of Mlc) (Fig. 4f). The second contact site involves interaction of the D-domain of the other Mlc protomer with MtfA near the putative catalytic cleft, burying an area of ~ 735 Å^2^. Both the stabilizing (α2) and the recognition helices (α3) of Mlc’s HTH motif interact with MtfA. Multiple salt bridges form between the guanidino groups of R33^B^ and R52^B^ and clustered carboxyl groups of aspartic acid residues D133, D136 and D139 (Fig. 4f), while hydrogen bonds between backbones of A43^B^/A199 and the side chain of N17^B^ with the backbone of N197 stabilize the complex. Interestingly, in this Mlc:MtfA complex, the two D-domains of Mlc are in close contact, forming an interaction between H9^A^ and Q41^B^ (not shown).

### Dynamics of regulatory Mlc complexes

**Fig. 5:**
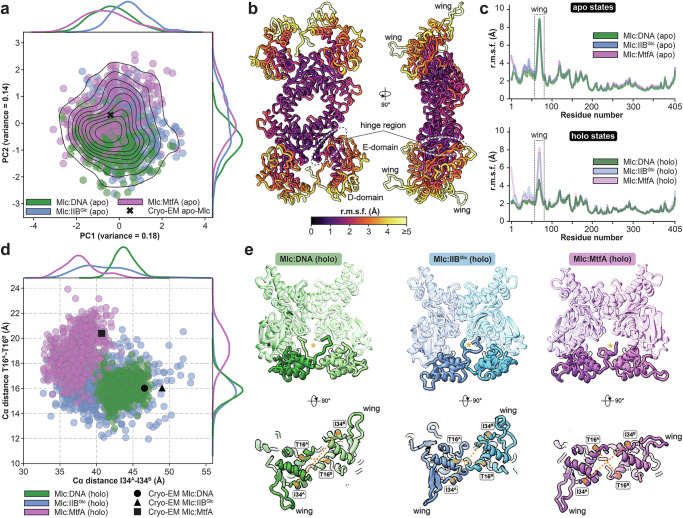
Dynamics of Mlc complexes analyzed by molecular dynamics (MD) simulations. **a** Principal component analysis (PCA) of apo-Mlc conformations from MD simulations initiated from holo-derived coordinates with interactors (DNA, IIB^Glc^ and MtfA) excluded. The experimentally determined apo-Mlc cryo-EM structure is indicated (black cross). Each point indicates a conformation of the corresponding Mlc, and the density is reported with contour lines. Marginal density distributions are shown for each principal component. **b** Visualization of tetrameric apo-Mlc dynamics, colored by average Cα root-mean-square fluctuations (r.m.s.f.). The different domains are indicated. **c** Per-residue Cα r.m.s.f. values for the Mlc protomer (averaged over all protomers and replicas) in apo and holo states reveals a stabilization of the wing upon DNA binding in Mlc:DNA. **d** Orientations of D-domains in Mlc:DNA, Mlc:IIB^Glc^ and Mlc:MtfA complexes are compared as a scatterplot of inter-residue distances T16^A^-T16^B^ and I34^A^-I34^B^. Starting configurations derived from the experimental structures are indicated. Superscript letters denote Mlc protomers. **e** Comparison of representative MD snapshots of Mlc dimers (D-domains highlighted) interacting with DNA, IIB^Glc^ and MtfA (interactors not shown for clarity). The orange asterisks mark the N-termini inserted into the dimer grooves. Different D-domain arrangements are shown (*bottom*), with distances between T16^A^-T16^B^ and I34^A^-I34^B^ indicated by dashed lines.

To gain insights into the dynamics of the Mlc complexes, we performed all-atom molecular dynamics simulations in three replicas, yielding a cumulative run-time of 23.8 µs (each ≥ 1.3 μs). For the Mlc:IICB^Glc^ complex, simulations were restricted to the Mlc:IIB^Glc^ moiety to reduce system size, as the distant IIC^Glc^ domain (Fig. 3a) is unlikely to affect core complex dynamics. First, we prepared models of apo-Mlc by removing its interaction partners (i.e., DNA, IIB^Glc^ and MtfA). All simulations converged to a structural ensemble consistent with the experimental apo-Mlc cryo-EM structure (Fig. 5a). To assess the overall dynamics of the repressor, we analyzed fluctuations across all apo-Mlc simulations. The structures remained stable throughout the simulations, with a mean root-mean-square fluctuation (Cα r.m.s.f.) of ~ 2 Å. The tetrameric core of Mlc, formed by the di- and tetramerized O-domains, remained the most stable, whereas the E-domains and, in particular, the D-domains exhibited considerable flexibility across all protomers (Fig. 5b and Supplementary Fig. 8a). These peripheral domains undergo coordinated rigid-body movements originating from a hinge region between the E- and O-domains. A comparable dynamic behavior was observed for the Mlc complexes when bound to interaction partners (holo-Mlc) (Supplementary Fig. 8b). Pronounced flexibility of the disordered wing domains was consistently observed in all apo states but was markedly reduced in the Mlc:DNA complex (Fig. 5c), consistent with the cryo-EM structure (Supplementary Fig. 8c). By contrast, the wings remain highly flexible in Mlc:IIB^Glc^ and Mlc:MtfA complexes (Fig. 5c). Next, global dynamic differences between the regulatory complexes were assessed by principal component analysis (PCA), comparing each holo state with its corresponding apo-state (Supplementary Fig. 8d). The overall dynamics were comparable for Mlc:DNA and Mlc:IIB^Glc^, with no clear difference identified, whereas Mlc:MtfA displayed distinct dynamics between holo and apo forms (Supplementary Fig. 8d). As a comparative measure, we defined two near perpendicular axes based on the Cα-Cα distances between T16^A^-T16^B^ and I34^A^-I34^B^ within the opposing HTH motifs of the dimer (Mlc protomers are denoted as A and B) (Fig. 5d). The relative orientation of the opposing HTH motifs at the dimer interface, described by the rhombus formed by these Cα-Cα distances, reveals clear conformational differences: a more equilateral arrangement in the Mlc:MtfA complex compared to the more elongated arrangement in Mlc:DNA and Mlc:IIBGlc complexes (Fig. 5e). Both DNA and MtfA protein, which contact the two D-domains, restrict interdomain flexibility, as reflected by the unimodal T16^A^-T16^B^ distances of ~19 and ~16 Å, respectively. In contrast, IIB^Glc^, which interacts laterally with the Mlc protomers, rigidifies the T16^A^-T16^B^ axis (average ~16 Å), but displays a near bimodal distribution of the I34^A^-I34^B^ axis with peaks at ~ 39 and ~ 44 Å (Fig. 5d). Another notable observation from the simulations concerns the N-termini of Mlc, which could not be modeled in the experimental cryo-EM maps due to uninterpretable density. In all modeled protomers, these N-terminal regions were found to partially insert in the cavity formed between the dimers (Fig. 5e), suggesting an additional stabilizing effect. This interpretation is supported by the disordered density observed within this cavity in unsharpened maps, consistent with a transient and/or heterogeneous interaction.

To assess how interfacial residues contribute to complex stability, we analyzed their evolution across the entire simulation trajectories and visualized them as heatmaps showing the frequency of polar interactions, i.e., hydrogen bonds and salt bridges (Supplementary Fig. 9). These analyses revealed that all major interfaces between Mlc and DNA, IIB^Glc^ and MtfA, and within the Mlc tetramer remained stable. Within the Mlc tetramer interface, the hydrogen bond between S287^A^ and D251^D^ displayed the highest frequency, followed by interactions between the backbone of G292^A^ and the side chains of N283^D^ and Q284^D^ (Supplementary Fig. 9a). The Mlc:DNA interface was characterized by several high-frequency contacts, particularly between positively charged and polar Mlc residues and the negatively charged DNA backbone (Supplementary Fig. 9d). In the Mlc:IIB^Glc^ complex, a backbone interaction between T186(O) in Mlc and V450(N) in IIB^Glc^ emerged as the primary binding contact (Supplementary Fig. 9c). In the Mlc:MtfA complex, both Mlc protomers stably contributed to the interface with MtfA. Especially R38^A^, R52^A^, and R73^A^ from Mlc strongly interact with the side chains of E210, D118 and D121, and E6 from MtfA, respectively, while the other Mlc protomer primarily interacts via its K49^B^ with E174 and E177 in MtfA (Supplementary Fig. 9b). Collectively, these dynamic interactions closely reflect those observed experimentally by cryo-EM.

## Discussion

The repressor Mlc of *E. coli* integrates environmental signals with transcriptional control over genes involved in carbohydrate utilization, thereby acting as a central regulator of cellular physiology. Unlike classical repressors that rely on small-molecule allosteric ligands, Mlc regulation emerges from a network of protein-protein and protein-DNA interactions, highlighting an alternative evolutionary strategy for metabolic sensing. In this study, we report the cryo-EM structures of Mlc in isolation, bound to a cognate DNA operator, and in complexes with the PTS glucose transporter IICB^Glc^ and the anti-repressor MtfA. Together with molecular dynamics simulations, these structures reveal a mechanism in which Mlc acts as a tetrameric scaffold whose activity on transcription is modulated by steric control through binding partners, rather than by conformational switching.

**Fig. 6:**
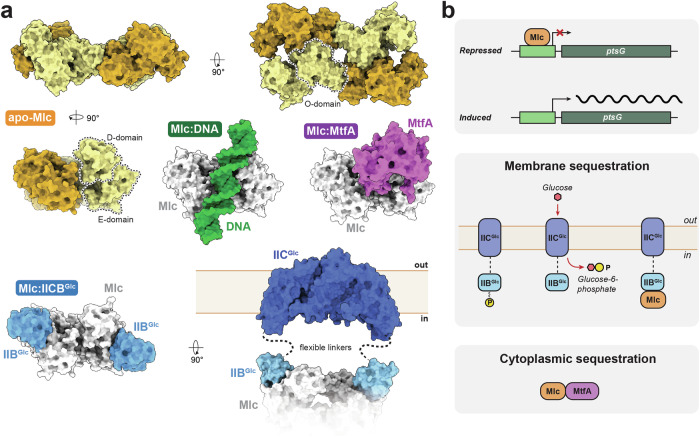
Structural comparison of Mlc regulatory states suggests a steric exclusion mechanism. **a** Overview of the tetrameric apo-Mlc structure shown as a surface representation (*top*), with protomers colored individually in yellow and orange. The central O-domains form a structural scaffold for the dimer-of-dimers assembly, while the peripheral E- and D-domains are capable of interacting with different interactors to regulate activity (representative domains in one protomer are contoured). A side-by-side structural comparison of Mlc (white surface) in complex with its interaction partners is shown. The DNA operator (green) and the anti-repressor MtfA (pink) compete for partially overlapping binding regions, whereas IIB^Glc^ proteins (light blue) bind laterally to the Mlc dimer. The relative position of the covalently connected, membrane-embedded IIC^Glc^ transporter dimer (dark blue) is indicated. **b** Schematic overview of the proposed Mlc-mediated regulatory pathways governing expression of the *ptsG* gene (dark green). Under glucose-limiting conditions, the Mlc repressor occupies its cognate operator site within the *ptsG* promoter (light green), thereby inhibiting transcription. In the presence of extracellular glucose, Mlc binds to dephosphorylated IICB^Glc^, leading to the relocation of Mlc to the cell membrane and resulting in the activation of *ptsG* transcription (de-repression)^18–24^. Alternatively, Mlc activity can be repressed through cytosolic sequestration by the anti-repressor MtfA^25–27^, similarly permitting *ptsG* expression.

One of the key findings of our study is the determination of the native oligomeric architecture and structure of Mlc. The homo-tetramer adopts a double X-shaped dimer-of-dimers assembly stabilized by tip-to-groove interactions between O-domains (Figs. 1, 6a). The structural elements mediating these tip-to-groove interactions are likely influenced and stabilized by the zinc finger motif, consistent with a previous report^28^. This tetrameric configuration mediated by the O-domains also proved central to the dynamics of Mlc, serving as a rigid scaffold while the peripherally located D- and E-domains retained flexibility for interactor engagement (Fig. 5b). Likely due to crystal packing effects and crystallization conditions in a previously reported crystal structure^24^, the tetramerization mode and interfaces in our Mlc structures are fundamentally different. Another crystal structure^28^, where tetramerization had been dismissed as an artifact, now turns out to be more plausible due to its similarity with the here reported in solution cryo-EM Mlc structure (Supplementary Fig. 3d). The apo-Mlc structure possibly reflects an intermediate state in transcription regulation without binding partners. When bound to its cognate operator, Mlc adopts an active repressor conformation that sterically blocks transcription initiation (Fig. 6b). DNA binding involves the two D-domains within the Mlc dimer, engaging in a near-symmetric manner with the pseudo-palindromic operator (Fig. 2). Neither the HTH nor the N-terminal region of Mlc form specific base contacts and rather mediates phosphate backbone interactions. Instead, the wings undergo a disorder-to-order transition (compared to apo-Mlc) and interact with the DNA’s minor groove (Fig. 2 and Supplementary Fig. 8b). Specifically, two arginine residues are positioned in the dA/dT-rich minor groove, which features a highly negative electrostatic potential^33^, and form the only observed base contacts (Fig. 2h). These wing interactions with the conserved, pseudo-palindromic sequence plausibly explain the major determinants of Mlc’s specificity and are consistent with previous reports, demonstrating the importance of conserved arginine residues for interactions within the operator’s minor groove^29,34^. The complexes of Mlc with IICB^Glc^ and MtfA revealed alternative mechanisms for de-repression (Figs. 3, 4). Their distinct localizations, i.e., membrane-associated via IICB^Glc^ versus soluble for MtfA, imply context-dependent mechanisms for sequestering Mlc (Fig. 6b). Particularly, the cognate operator DNA and MtfA bind to overlapping interaction surfaces on Mlc, suggesting that their binding is mutually exclusive (Fig. 6a). For MtfA, the interface observed in the cryo-EM structure is consistent with prior work identifying key contributions of MtfA to Mlc binding through mutagenesis and functional reporter assays^26^. Conversely, the IIB^Glc^ domains of the IICB^Glc^ transporter bind peripherally to the DNA interface of Mlc and thus do not compete for the same interface. However, Mlc membrane localization through IICB^Glc^, and thus sequestration of the Mlc repressor at the membrane^23,24^, plausibly imposes a localization-dependent constraint on Mlc activity by limiting its access to DNA targets. The structure of nearly complete IICB^Glc^ provides insights into how the flexible and mobile IIB^Glc^ domains participate in regulatory functions. The disordered linker connecting IIC^Glc^ and IIB^Glc^, allows IIB^Glc^ to reposition for phosphorylation of translocated glucose and to dynamically engage Mlc at the membrane.

We show that apo-Mlc is competent to interact with all its partners, underscoring its regulation through protein-protein and protein-DNA interactions. Complex formation induces distinct structural and dynamic changes in Mlc depending on the interacting partner. Binding to DNA, for instance, triggers a disorder-to-order transition in the wing region of Mlc and induces local distortions in the DNA itself, while the binding to MtfA alters the dynamics of the D-domains. As a common important feature, the interactions between Mlc and its partners are mediated by complementary electrostatic surfaces, with positively and negatively charged regions engaging each other. MD simulations further provided critical insights into the temporal dynamics and allowed identification of the major contributors for these transient interactions, explaining the stability of the macromolecular assemblies. While our data support the canonical oligomeric assembly of Mlc, hetero-complexes involving simultaneous DNA binding and protein-protein interactions are structurally conceivable. Such assemblies could add an additional layer of regulatory complexity, yet their structural and biological significance remains to be elucidated.

Overall, our findings advance the mechanistic understanding of bacterial transcriptional regulation and exemplifies Mlc regulation as an alternative to classical small-molecule induction. By elucidating the assembly of Mlc and its structural adaptability in regulatory complexes at the molecular level, this work lays the foundation for future studies on regulatory interplay and may inspire broader exploration of protein sequestration strategies in prokaryotic gene regulation.

## Methods

### Construct design and cloning

A modified version of the pTrcHisA plasmid (Addgene) was prepared as follows: The HindIII restriction site in the multiple cloning site was silenced, and a human rhinovirus (HRV) 3C protease cleavage site (followed by a new HindIII restriction site) was inserted using BamHI and BglII, yielding the empty H_6_-3C-pTrc plasmid. The *mlc* gene, encoding the Mlc protein (UniProt ID: P50456) from *E. coli* K-12, was amplified from genomic DNA, and the isolated PCR products were digested with the restriction enzymes HindIII and XhoI. Finally, the insert was ligated into the H_6_-3C-pTrc plasmid using HindIII and XhoI restriction sites, yielding an N-terminally hexa-histidine-tagged (H_6_) construct separated by a 3C cleavage site (H_6_-3C-Mlc). The *mtfA* gene encoding MtfA (UniProt ID: P76346) was analogously amplified from genomic DNA and ligated into the pZUDF plasmid^35^ using HindIII and XhoI restriction sites, yielding a C-terminally deca-histidine-tagged (H_10_) construct separated by a 3C protease cleavage site (MtfA-3C-H_10_). All sequences were verified by gene sequencing (Microsynth).

### Overexpression and purification of Mlc

The plasmid encoding Mlc was transformed into *E. coli* BL21(DE3) BW25113 Δ*acrB*Δ*ptsG* cells^31^. For overexpression, Luria-Bertani (LB) medium supplemented with 100 μg/mL ampicillin was inoculated with an overnight pre-culture (1:100 ratio) and cultivated at 180 rpm at 37 °C in an incubator shaker (Multitron, Infors HT) until the culture reached an optical density of 0.9 at 600 nm (OD_600_). Prior induction with 200 µM isopropyl-β-D-thiogalactopyranoside (IPTG, final concentration) and expression at 18 °C, culture flasks were cooled down for 30 min in an ice bath and supplemented with 50 mL/L ethanol (EtOH) and 50 µM ZnCl_2_ (Sigma). The following day, cells were harvested by centrifugation (10,000 × *g*, 10 min, 4 °C), washed once in lysis buffer (50 mM glycine-NaOH pH 9.0, 300 mM NaCl, 1 mM β-ME (β-mercaptoethanol)) and stored as a pellet at − 80 °C. All subsequent purification steps were performed at 4 °C. Thawed cells were resuspended in lysis buffer containing 200 µg/mL lysozyme and 50 µM ZnCl_2_, and lysed by five passes through an M-110P Microfluidizer (Microfluidics) operated at 1500 bar. Cell debris was removed by consecutive low-speed (15,000 × *g*, 10 min, 4 °C) and high-speed (150,000 × *g*, 30 min, 4 °C) centrifugation steps. The resulting supernatant was then treated with 0.01% (v/v) branched polyethylenimine (M_W_ ~ 6000 Da) followed by an additional high-speed centrifugation step. Mlc residing in the supernatant was supplemented with 10 mM imidazole and 25 µg/mL DNase I, bound to Ni^2+^-NTA resin (Qiagen), washed with 20 column volumes of lysis buffer supplemented with 20 mM imidazole and eluted with lysis buffer supplemented with 300 mM imidazole. The purified protein was concentrated using an Amicon Ultra-4 centrifugal filter unit with a 100 kDa molecular weight cut-off (Millipore) to ~ 20 mg/mL and dialyzed overnight against lysis buffer in the presence of home-made HRV 3C protease ( ~ 0.4 mg/mL) to remove the hexa-histidine tag. The protein was then centrifuged (150,000 × *g*, 30 min, 4 °C) and snap-frozen in aliquots for storage at − 80 °C.

### Overexpression and purification of IICB^Glc^

The glucose-specific transporter IICB^Glc^ (UniProt ID: P69786) from *E. coli* was prepared essentially as described previously^31,36^. Briefly, the IICB^Glc^-3C-H_10_ construct encoded in the pZUDF plasmid was overexpressed in *E. coli* BL21(DE3) BW25113 Δ*acrB*Δ*ptsG* cells grown in LB medium supplemented with 100 μg/mL ampicillin, and overexpression was induced with 200 µM IPTG (final concentration) for 4 h. Harvested cells were lysed using a Microfluidizer as described above, cell debris were removed (15,000 × *g*, 10 min, 4 °C), and membranes were isolated by high-speed centrifugation (150,000 × *g*, 30 min, 4 °C). Membrane pellets were washed once by homogenization in ice-cold membrane buffer (40 mM 4-(2-hydroxyethyl)-1-piperazineethanesulfonic acid (HEPES)-NaOH pH 8.0, 300 mM NaCl), followed by centrifugation. The final pellets were then homogenized in membrane buffer at 200 mg/mL (w/v) and stored at − 80 °C. The subsequent detergent solubilization and purification steps were conducted at 4 °C: IICB^Glc^ was solubilized in the presence of 2% (w/v) *n*-dodecyl-β-D-maltopyranoside (DDM, Glycon) and 5 mM D-glucose (Sigma, final concentration), and the detergent insoluble material was removed by high-speed centrifugation. The supernatant was diluted (1:1) with wash buffer (20 mM HEPES-NaOH pH 8.0, 300 mM NaCl, 5 mM β-ME, 0.02% (w/v) DDM, 5 mM D-glucose and 20 mM imidazole) bound to Ni^2+^-NTA resin for 90 min, washed with 40 column volumes of wash buffer, and eluted in SEC buffer (20 mM HEPES-NaOH pH 8.0, 100 mM NaCl, 5 mM β-ME, 0.02% (w/v) DDM, 5 mM D-glucose) supplemented with ~ 0.4 mg/mL HRV 3C protease for on-column cleavage^37^. The eluted protein was concentrated in an Amicon Ultra-4 centrifugal filter unit with a 100 kDa molecular-weight cut-off and further purified by size-exclusion chromatography (SEC) using a Superdex 200 increase 10/300 GL column mounted on a ÄktaPure chromatography system (Cytiva) equilibrated with SEC buffer. Peak fractions containing IICB^Glc^ were pooled and immediately used for complex reconstitution with Mlc.

### Overexpression and purification of MtfA

The plasmid encoding MtfA was transformed into *E. coli* BL21(DE3) BW25113 Δ*acrB*Δ*ptsG* cells. For overexpression, LB medium supplemented with 100 μg/mL ampicillin was inoculated with an overnight pre-culture (1:100 ratio) and incubated at 37 °C with shaking at 180 rpm until the culture reached an OD_600_ of 1.2. Protein overexpression was induced by adding 500 µM IPTG (final concentration) and 50 µM ZnCl_2_, and incubation was continued overnight at 24 °C. After 18 h, cells were harvested by centrifugation (10,000 × *g*, 10 min, 4 °C), washed once in lysis buffer (50 mM Tris-HCl pH 8.0, 300 mM NaCl, 1 mM β-ME) and stored as a pellet at − 80 °C. All subsequent purification steps were performed at 4 °C. For purification of MtfA, cells were resuspended in lysis buffer (supplemented with 10 mM imidazole and 50 µM ZnCl_2_) lysed using a Microfluidizer, and cell debris were removed by sequential low- and high-speed centrifugation as described above. The protein in the supernatant was bound to Ni^2+^-NTA resin, washed with 30 column volumes of lysis buffer containing 20 mM imidazole, followed by 10 column volumes containing 40 mM imidazole and eluted with lysis buffer supplemented with 300 mM imidazole. The eluted protein was dialyzed against lysis buffer supplemented with 50 µM ZnCl_2_ overnight in the presence of home-made HRV 3C protease ( ~ 0.4 mg/mL) to cleave off the deca-histidine tag. The protein was then centrifuged at high speed (150,000 × *g*, 30 min, 4 °C) and the His-tagged HRV 3C protease was removed by a second incubation with Ni^2+^-NTA resin. MtfA in the flow-through was concentrated using an Amicon Ultra-4 centrifugal filter unit with a 10 kDa molecular weight cut-off to ~ 12 mg/mL and subjected to SEC on a Superdex 200 increase 10/300 GL column equilibrated with buffer (20 mM HEPES-NaOH pH 8.0, 150 mM NaCl, 5% (v/v) glycerol, 1 mM β-ME and 5 µM ZnCl_2_). Peak fractions were pooled, snap-frozen in liquid nitrogen and stored in aliquots at − 80 °C.

### Sample preparation for cryo-EM

For determination of the apo-Mlc structure, an aliquot was thawed on ice and further purified by SEC using a Superose 6 3.2/300 column mounted on an ÄktaPure device (Cytiva). The protein was eluted in buffer containing 20 mM HEPES-NaOH pH 8.0, 150 mM NaCl, 1 mM β-ME, 0.02% (w/v) DDM and 50 µM ZnCl_2_, and peak fractions were collected. For determination of the DNA operator-bound Mlc structure, the complex was assembled using apo-Mlc and a self-complementary DNA duplex covering the *ptsG*^*op*^ P1 promoter plus three additional bases pairs on each side. The duplex was formed by annealing the template strand 5’-GAATTATTTTACTCTGTGTAATAAATAAA-3’ with the complementary non-template strand 5’-TTTATTTATTACACAGAGTAAAATAATTC-3’. The commercially synthesized, desalted oligonucleotide (Integrated DNA Technologies) was rehydrated at a final concentration of 10 mg/mL (0.56 mM) in complex buffer (20 mM HEPES-NaOH pH 8.0, 150 mM NaCl, 1 mM β-ME, 1 mM MgCl_2_, 50 µM ZnCl_2_), heated to 42 °C for 10 min and slowly cooled down. Mlc was buffer-exchanged by SEC using a Superdex 200 5/150 column equilibrated with complex buffer. The complex was reconstituted by mixing Mlc with a slight excess of oligonucleotide at a 1:1.1 molar ratio (Mlc_dimer_:DNA duplex) and incubating the mixture for 30 min on ice. Analytical SEC was performed with a 25 µL aliquot while monitoring absorbance at 280 and 260 nm. The Mlc:IICB^Glc^ complex was assembled by mixing concentrated Mlc and IICB^Glc^ in a 1:1.05 molar ratio (Mlc_dimer_:IICB^Glc^_dimer_). After incubation on ice for 30 min, the sample was centrifuged (20,000 x *g*, 10 min, 4 °C) and injected into a Superdex 200 increase 10/300 GL column equilibrated with SEC buffer (as used for the purification of IICB^Glc^). Collected fractions were analyzed by SDS-PAGE, and the complex was concentrated to 5 mg/mL using an Amicon Ultra-0.5 100 kDa spin column concentrator (Millipore). The Mlc:MtfA complex was assembled by mixing Mlc and an excess of MtfA in a 1:1.25 molar ratio (Mlc_dimer_:MtfA_monomer_) and incubation for 1 h on ice. The complex was then isolated by preparative SEC using a Superdex 200 5/150 column equilibrated with a buffer containing 20 mM HEPES-NaOH pH 8.0, 150 mM NaCl, 1 mM β-ME, 5 µM ZnCl_2_, and the peak fractions were pooled.

### Cryo-EM grid preparation and data acquisition

Specimens for cryo-EM were prepared using a Vitrobot Mark IV (Thermo Fisher Scientific) operated at 4 °C and 100% humidity. Samples (3 μL each; previously centrifuged at 20,000 × *g*, 10 min, 4 °C) were applied to glow-discharged (15 mA, 0.25 mbar for 22.5 s) Quantifoil R1.2/1.3 or R2/1 200-mesh copper grids at the following concentrations: 1.25 mg/mL for apo-Mlc, 0.8 mg/mL for Mlc:DNA, 5 mg/mL for Mlc:IICB^Glc^ and 1.5 mg/mL for Mlc:MtfA. Grids were incubated for 10 s, blotted for 4 s with a blot force of -4 and vitrified by plunging into liquid ethane cooled by liquid nitrogen. Grids were screened on a 200 kV Tecnai F20 transmission electron microscope (FEI) equipped with a Falcon 3 direct electron detector.

High-resolution data collection for apo-Mlc, Mlc:DNA and Mlc:MtfA were performed on a Titan Krios G4 operated at 300 kV, equipped with a Falcon 4i direct electron detector (Thermo Fisher Scientific) and Selectris energy filter (set to 10 eV). Movies in counting mode (in the .eer format) were collected using EPU at 165,000× (apo-Mlc) or 130,000 × (Mlc:DNA and Mlc:MtfA) magnification over a defocus range of − 0.8 to − 2.2 µm and physical pixel sizes of 0.7328 Å, 0.9376 Å and 0.9374 Å, amounting to total doses between 30 and 35 e^-^/Å^2^. High-resolution data collection for Mlc:IICB^Glc^ was performed on a Titan Krios G2 operated at 300 kV, equipped with a K3 Summit direct electron detector (Gatan) and Quantum energy filter (set to 20 eV). Movies in counting mode (in the .tif format) were collected by dose fractionation in 40 frames using SerialEM^38^ at 105,000 × magnification over a defocus range of − 0.7 to − 1.7 µm and a physical pixel size of 0.8220 Å, amounting to a total dose of 50.30 e^-^/Å^2^.

### Cryo-EM image processing

Single-particle analysis data processing was performed using the software package cryoSPARC^39^ (multiple versions). Processing and validation details are provided below and in Supplementary Figs. 1, 5, 6 and 7, and in Supplementary Data 1. Generation of composite maps for the Mlc complexes is described in Supplementary Fig. 2.

#### apo-Mlc structure

A total of 6521 movies were imported and subjected to patch CTF estimation and motion correction. Micrographs with poor statistics were discarded, resulting in 6106 micrographs. References for template picking were generated from manually picked coordinates, and ultimately 2,571,849 particles were automatically picked and extracted (box size of 400 pixel (px), binned to 96 px). Four consecutive rounds of two-dimensional (2D) classification (60 iterations, uncertainty factor = 8, 4 full iterations) were used to remove bad picks, and the remaining 1,033,278 particles were used for parallel multi-reference ab-initio reconstruction. Particles belonging to good classes were pooled and duplicates within a distance < 80 Å were removed, and the procedure was repeated. This yielded the final particle stack comprising 339,926 particles, which were re-extracted (box size of 440 px, binned to 320 px) and aligned using homogenous and non-uniform refinements. Local and global CTF refinements were performed, and a soft mask enclosing protein density was generated in ChimeraX v1.4^40^ using the *molmap* command and used for consecutive rounds of local refinement (with finer search criteria in each round) with C_2_-symmetry imposed. This yielded the consensus three-dimensional (3D) reconstruction with a global resolution of 2.74 Å at Fourier-shell correlation (FSC) = 0.143. To enhance the resolution, local refinements were performed using masks encompassing either the oligomerization domains or the peripheral regions, followed by focused refinement of C_2_-symmetry-expanded particles. Particles for the peripheral parts underwent an additional round of 3D classification without alignment, yielding 244,307 particles. The final local refinements yielded focused reconstructions with global resolution of 2.62 and 2.89 Å at FSC = 0.143. The density half-maps of globally and locally refined volumes were post-processed using the DeepEMhancer Python script (v3.1)^41^ with the *highRes* learning model. The composite map for apo-Mlc was generated in ChimeraX as follows (Supplementary Fig. 2a): One copy of focused map 1 and two copies of focused map 2 were aligned onto the consensus map (Supplementary Fig. 1a, d). The three aligned focused maps were then combined into a single map (i.e., the composite map) using the *vop maximum* command in ChimeraX. 3D variability analysis^42^ (3DVA) was performed using the particles contributing to the consensus refinement, applying a 5 Å filter resolution and solving for three components. The resulting components were analyzed using the built-in clustering mode.

#### Mlc:DNA structure

A total of 10,993 movies were imported and subjected to patch-based CTF estimation and motion correction. Micrographs with poor statistics were discarded, resulting in 9939 micrographs. References for template picking were generated from manually picked coordinates, and ultimately 2,199,177 particles were automatically picked and extracted (box size of 400 px, binned to 96 px). Two consecutive rounds of 2D classification (60 iterations, uncertainty factor = 8, 4 full iterations) were used to remove bad picks and the remaining 948,000 particles showing secondary structure features were used for three parallel multi-reference ab-initio reconstructions (requesting each three classes). Particles belonging to good classes were pooled and duplicates within a distance < 80 Å were removed. Subsequently, homogenous and heterogeneous refinements were performed, yielding a particle stack comprising 322,270 particles. These particles were re-extracted (box size of 320 px, no binning) and subjected to further homogenous and non-uniform refinements. Local and global CTF refinements were then performed, followed by the generation of a soft mask encompassing the complex density. This mask was used for three successive rounds of local refinement with progressively finer search parameters, and C_2_-symmetry imposed, yielding a consensus 3D reconstruction at a global resolution of 2.47 Å at FSC = 0.143. To improve the interpretability of peripheral densities, particles were C_2_-symmetry-expanded and subjected to a 3D classification without alignment, followed by local refinements using a soft mask covering half of the complex. The final particle stack comprised 248,583 particles and produced a focused map with a global resolution of 2.56 Å at FSC = 0.143. Density half-maps were post-processed using the DeepEMhancer Python script with the *highRes* learning model. The composite map for Mlc:DNA was generated in ChimeraX as follows (Supplementary Fig. 2b): Two copies of the focused map were aligned onto the consensus map (Supplementary Fig. 5b, e). The two aligned focused maps were then combined into a single map (i.e., the composite map) using the *vop maximum* command in ChimeraX.

#### Mlc:IICB^Glc^ structure

A total of 19,806 movies were imported and subjected to patch CTF estimation and motion correction. Micrographs with poor statistics were discarded, resulting in 13,890 micrographs. Initial processing revealed considerable conformational heterogeneity within the complex, prompting the use of a specialized processing strategy. The complex was divided into two regions for separate processing: the central moiety (corresponding to Mlc:IIB^Glc^) and the peripheral moiety (corresponding to IIC^Glc^). Template-picking references were generated from manually selected particles for each region. For the central moiety, 6,059,378 particles were picked and extracted (box size of 360 px, binned to 96 px), whereas for the peripheral moiety, 10,903,262 particles were automatically picked and extracted (box size of 220 px, binned to 60 px). In both cases, consecutive rounds of 2D classification were performed, followed by various 3D classifications and refinements (i.e., ab-initio reconstructions, homogenous, heterogenous and non-uniform refinements) and re-extraction of selected particles (box size of 480 px, binned to 320 px for Mlc:IIB^Glc^; box size of 360 px, binned to 300 px for IIC^Glc^). Further 3D classification and refinement (i.e., local and global CTF refinements, local and non-uniform refinements) yielded the final particle stacks, comprising 406,110 particles for Mlc:IIB^Glc^ and 603,986 particles for IIC^Glc^. Specifically, the peripheral region was refined with C_2_-symmetry imposed using local refinement and a soft mask encompassing the IIC^Glc^ moiety, resulting in a 3D reconstruction with a global resolution of 2.81 Å at FSC = 0.143. The Mlc:IIB^Glc^ map obtained after non-uniform refinement featured densities for the peripheral part at lower thresholds and was thus used as the consensus map. To further enhance interpretability, these particles were C_2_-symmetry-expanded and subjected to focused local refinement, resulting in maps with global resolutions of 2.78 Å and 2.95 Å at FSC = 0.143. Finally, the density half-maps were used for post-processing using the DeepEMhancer Python script with the *highRes* learning model. The composite map for Mlc:IICB^Glc^ was generated in ChimeraX as follows (Supplementary Fig. 2c): One copy of focused map 1, two copies of focused map 2 and two copies of focused map 3 were aligned onto the consensus map (Supplementary Fig. 6f, g). The aligned focused maps were then combined into a single map (i.e., the composite map) using the *vop maximum* command in ChimeraX.

#### Mlc:MtfA structure

A total of 13,208 movies were imported and subjected to patch CTF estimation and motion correction. Micrographs with poor statistics were discarded, resulting in 10,780 micrographs. References for template picking were generated from manually picked coordinates, and ultimately, 3,893,353 particles were automatically picked and extracted (box size of 360 px, binned to 96 px), followed by three consecutive rounds of 2D classification yielding 1,469,021 particles. Ab-initio reconstruction and homogeneous refinement were performed, and particles were re-extracted (box size of 360 px, binned to 300 px) and subjected to non-uniform refinement to obtain a consensus 3D reconstruction with a global resolution of 2.30 Å at FSC = 0.143. Despite the seemingly high resolution, the peripheral regions remained poorly defined. Therefore, particles were C_2_-symmetry-expanded and subjected to two rounds of 3D classification without alignment, followed by local refinements using soft masks covering either the central or peripheral regions of the complex. The final local refinements yielded focused reconstructions with global resolutions of 2.30 (578,981 particles) and 2.94 Å (251,059 particles) at FSC = 0.143. Each map was post-processed using the DeepEMhancer Python script with the *highRes* learning model. The composite map for Mlc:MtfA was generated in ChimeraX as follows (Supplementary Fig. 2d): One copy of focused map 1 and two copies of focused map 2 were aligned onto the consensus map (Supplementary Fig. 7b, e). The three aligned focused maps were then combined into a single map (i.e., the composite map) using the *vop maximum* command in ChimeraX.

### Model building, refinement and validation

As an initial template for model building, we used the AlphaFold-predicted structure of Mlc from *E. coli* K-12 (AF-P50456). The model was fitted into the DeepEMhancer-post processed cryo-EM composite map of apo-Mlc using the interactive molecular-dynamics flexible fitting function in ISOLDE^43^, implemented as a plugin in ChimeraX. The tetrameric model was then obtained by fitting the adjusted protomer models into the map. Several rounds of manual refinement were performed in Coot^44^ (version 0.9.8), followed by iterative *real_space_refinement* in Phenix^45,46^ v1.20.1 until satisfactory statistics were achieved. Model quality was validated using MolProbity^47^ within Phenix. Models for the other complexes were built and validated using comparable procedures based on their respective composite maps. An initial model for double-stranded B-form DNA was generated in ChimeraX with the *build start nucleic* command, using the DNA sequence provided above as input. Initial models for the protein interactors were obtained as follows: The AlphaFold-predicted structures from *E. coli* K-12 were used for IIB^Glc^ (AF-P69786) and MtfA (AF-P76346), while the model for IIC^Glc^ was derived from PDB ID 8QSR^31^. Restraints for the modeled glucose molecules were generated using eLBOW^48^. Representative cryo-EM densities with fitted models and model-to-map FSC plots (at FSC = 0.5) are provided (Supplementary Fig. 10).

### Molecular dynamics simulations

Molecular dynamics (MD) simulations were performed to investigate the conformational dynamics of the transcription factor Mlc in both apo and holo states, starting from its experimentally determined structures in complex with DNA, IIB^Glc^ or MtfA, resulting in six distinct systems (Supplementary Table 1). Since the IIB^Glc^- and MtfA-bound structures lacked portions of the N-terminal and wing loop regions, these missing segments were modeled using the more complete DNA-bound structure as a template. The AMBER force field ff14SB^49^ was used to parameterize the protein components, with the OL15^50^ modification applied in systems containing DNA. Zn^2+^-binding sites in Mlc and MtfA were modeled as covalent interactions using the Zinc AMBER Force Field (ZAFF)^51^, following the ZAFF modeling tutorial (https://ambermd.org/tutorials/advanced/tutorial20/ZAFF.php). All systems were solvated using the TIP3P water model^52^ and neutralized with NaCl. Simulations were carried out using OpenMM 7.7.0^53^. Electrostatic interactions were computed using the Particle Mesh Ewald (PME) method. Van der Waals interactions were treated with force switching, employing a switching distance of 10 Å and a cut-off at 12 Å. Langevin dynamics with a friction coefficient of 1 ps^-1^ maintained the temperature at 303.15 K, and pressure was controlled at 1.0 bar using the Monte Carlo barostat^54^. Each system was energy-minimized for 5000 steps, followed by a 2000 ps equilibration phase during which sidechain restraints were removed. All restraints were lifted in the subsequent production run. A 2-fs integration time step was used for all simulations. Each system was simulated in triplicate, yielding 18 independent trajectories. Simulations were run up to 1.3 μs per replica; two simulations (Mlc:DNA (apo), replica 3 and Mlc:MtfA (apo), replica 1) were extended to 1.5 μs to ensure convergence based on Cα r.m.s.d. analysis (Supplementary Fig. 11). The total cumulative simulation time amounted to 23.8 μs. Trajectory analysis was performed using VMD 1.9.4^55^ in combination with in-house Tcl scripts and Python scripts using ProDy 2.5^56–58^. Hydrogen bond occupancies were calculated using the VMD HBonds Plugin^55^, applying a distance cut-off of 4 Å and an angle cut-off of 30°.

### Miscellaneous

The purity of the proteins was assessed by SDS-PAGE using 13.5% (w/v) SDS/polyacrylamide gels stained with Coomassie Brilliant Blue R-250. The Precision Plus Protein™ Dual Color Standard (BioRad) was used as a molecular weight marker. SEC columns were calibrated with the high molecular weight calibration kit (Cytiva). Density maps and models were visualized and rendered in ChimeraX, and figures were prepared in Adobe illustrator (Adobe Inc). Molecular interfaces were analyzed using the PDBePISA server (http://www.ebi.ac.uk/pdbe/pisa). Structural alignments were performed in ChimeraX using the *matchmaker* command, and the IIC^Glc^ protein domain was positioned within the membrane using the PPM server^59^. The sequence logo was prepared using the WebLogo server^60^ based on *E. coli* K-12 (MG1655) Mlc operator sequences (Source Data). DNA helical parameters were calculated using the Web 3DNA 2.0 server^61^, and the major and minor groove widths (defined as distances between the closest interstrand backbone phosphates) were calculated by subtracting 5.8 Å to account for the van der Waals radii of the phosphates. Per-residue conservation analysis was performed using the ConSurf algorithm, executed via both the web server (https://consurf.tau.ac.il) and the Google Colab implementation provided by the developers (used due to temporary unavailability of the server). Analyses were conducted based on the full-length protein sequences (UniProt ID codes: P50456, P76346 and P69786 for Mlc, MtfA and IICB^Glc^, respectively) using default parameters. Briefly, sequences of homologs were identified and filtered to retain sequences within 35–95% identity. A maximum of 150 non-redundant sequences was automatically selected using the *sample* mode to reduce redundancy bias. Multiple sequence alignment was performed with MAFFT, and conservation scores were calculated using the empirical Bayesian method.

### Reporting summary

Further information on research design is available in the Nature Portfolio Reporting Summary linked to this article.

## Supplementary information

Supplementary Information

Description of Additional Supplementary Files

Supplementary Data 1

Reporting Summary

Transparent Peer Review file

## Source data

Source Data

## Data Availability

Structural model coordinates and cryo-EM maps have been deposited in the Protein Data Bank (PDB, https://www.rcsb.org/) and Electron Microscopy Data Bank (EMDB, https://www.ebi.ac.uk/emdb/). The primary maps (i.e., the composite maps) and associated molecular models were deposited with the following accession codes: 9SRT and EMD-55153 (apo-Mlc), 9SRU and EMD-55156 (Mlc:DNA complex), 9SRX and EMD-55165 (Mlc:IICB^Glc^ complex), and 9SRW and EMD-55160 (Mlc:MtfA complex). Secondary EMD accession codes for consensus and focused reconstructions are: EMD-55150, EMD-55151 and EMD-55152 (apo-Mlc); EMD-55154 and EMD-55155 (Mlc:DNA); EMD-55161, EMD-55162, EMD-55163 and EMD-55164 (Mlc:IICB^Glc^); EMD-55157, EMD-55158 and EMD-55159 (Mlc:MtfA) - see Supplementary Data 1 for detailed information. PDB accession codes used in this study for comparison are: 1Z6R, 3BP8, 3BP3 and 1O2F. Initial and final frames of the MD simulations are available at Zenodo (10.5281/zenodo.17159479)^62^. Source data are provided in this paper.

## Code availability

The code used for MD analysis is available at GitHub (https://github.com/ibmm-unibe-ch/Mlc)^63^.
