# Supplementary material for: Structural basis of Mlc-mediated transcriptional regulation of carbohydrate metabolism
Source: Nat Commun. 2026 Jul 11;17:8553. doi: 10.1038/s41467-026-75270-8 (PMC13482132; doi:10.1038/s41467-026-75270-8)
Supplement: Supplementary file 1 [file 41467_2026_75270_MOESM1_ESM.pdf]

Supplementary Information

Structural basis of Mlc-mediated transcriptional regulation of carbohydrate metabolism

Patrick Roth, Inken Fender, Jean-Marc Jeckelmann, Zöhre Ucurum, Thomas Lemmin and Dimitrios Fotiadis*

Institute of Biochemistry and Molecular Medicine, Medical Faculty, University of Bern, Bern, Switzerland

* Corresponding author: dimitrios.fotiadis@unibe.ch

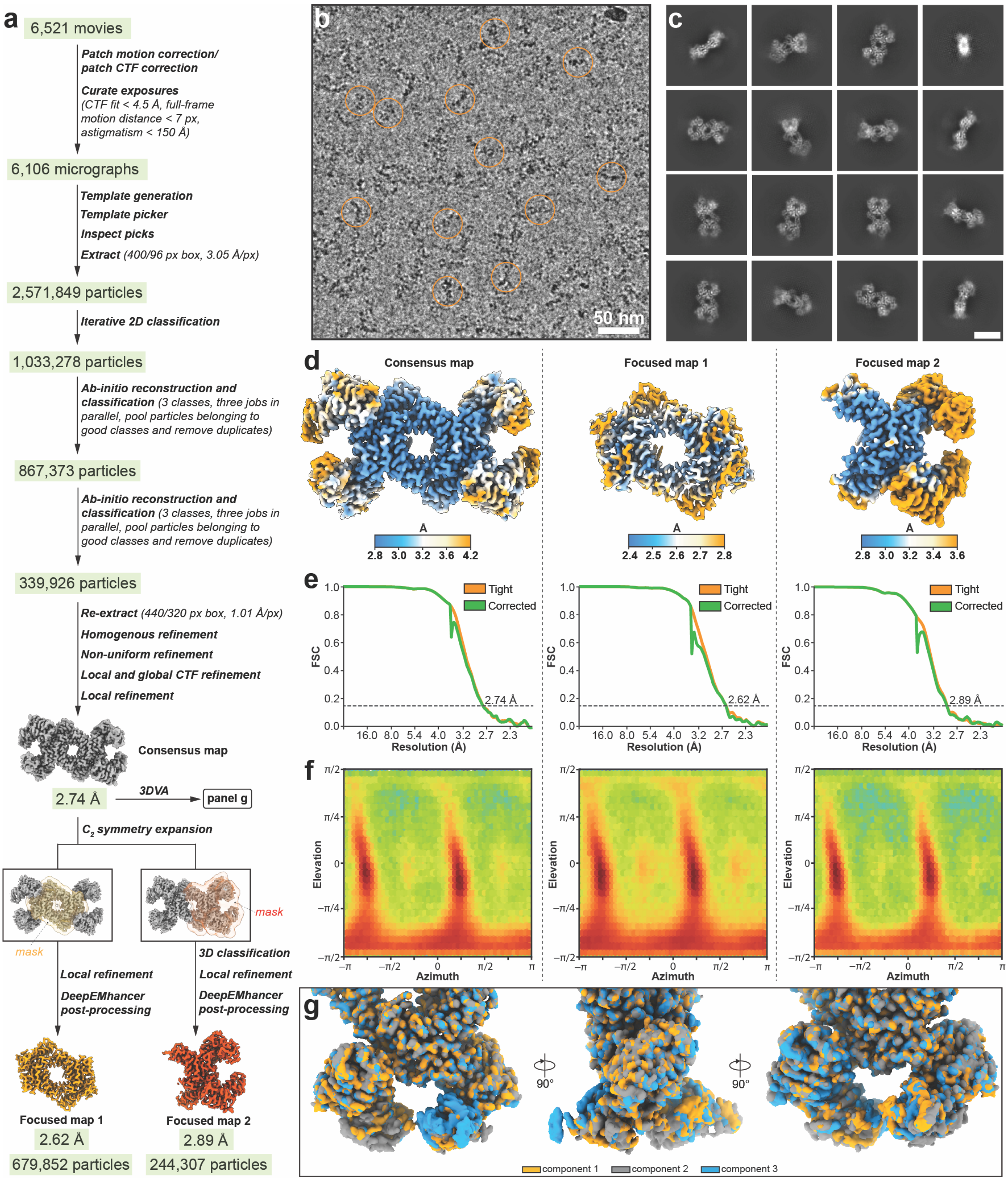

Supplementary Fig. 1: Cryo-EM single-particle analysis, 3D reconstruction and validation of apo-Mlc. (**a**) Data processing workflow. The soft masks (transparent surfaces) used for focused classification and local refinement are indicated (box). Further details on data processing are provided in the methods section. (**b**) Exemplary electron micrograph (at 2.1 µm defocus, low pass filtered) with representative particles highlighted by orange circles. The scale bar represents 50 nm. (**c**) Representative 2D class averages. The scale bar represents 10 nm. (**d**) Cryo-EM density maps: consensus and locally refined focused maps colored by computed local resolutions. Color bars indicate resolution in Å. (**e**) Fourier-shell correlation (FSC) plots. (**f**) Viewing-direction distribution plot showing the number of particles at specific elevation and azimuth angles contributing to the final reconstructions. Red means more and blue fewer particles of the specific viewing directions. (**g**) 3D variability analysis (3DVA) components reveal conformational flexibility at the protein's periphery. The corresponding maps are shown from three different perspectives.

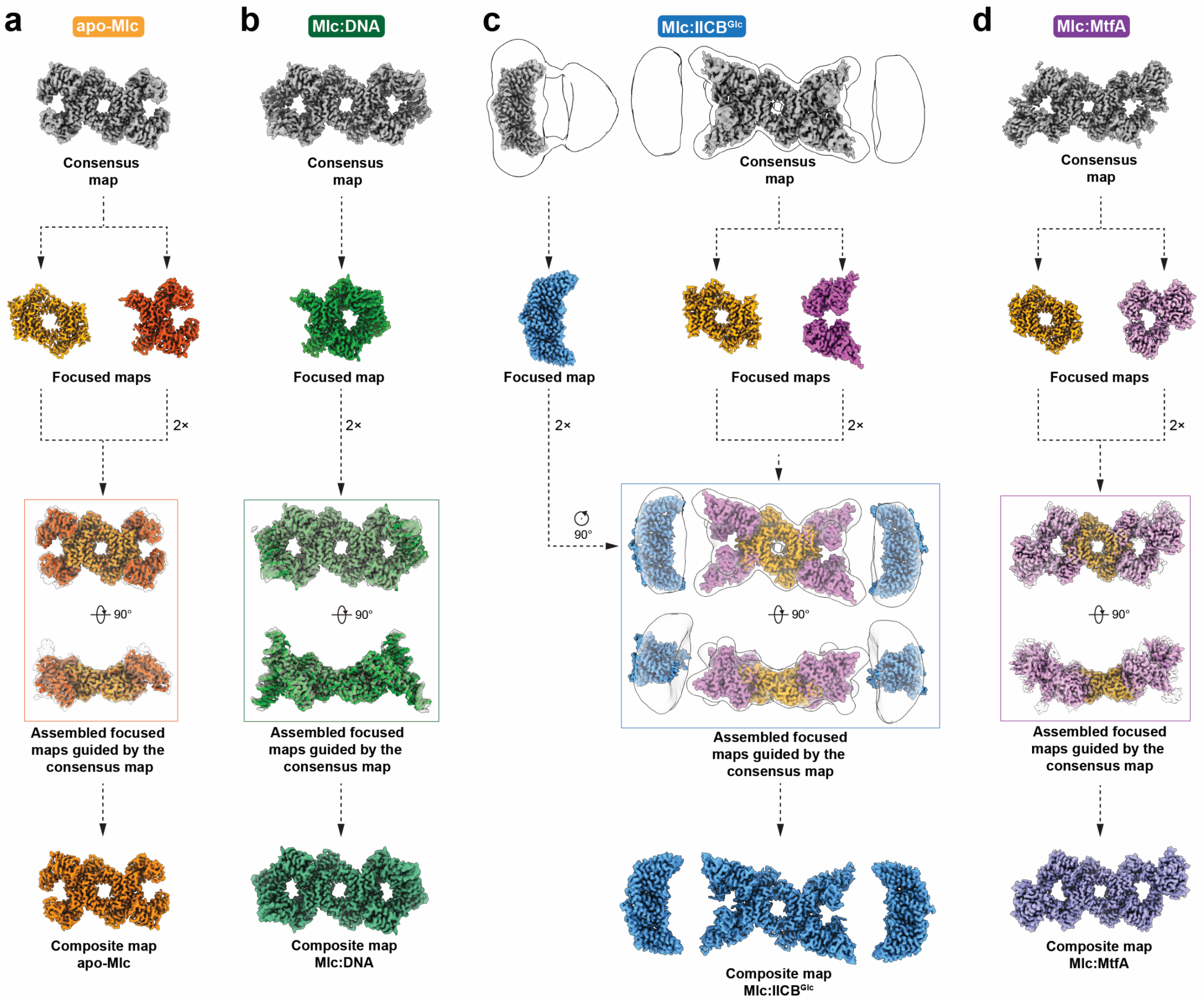

Supplementary Fig. 2: Generation of composite maps for the different Mlc complexes. To account for conformational flexibility in the Mlc complexes, initial consensus reconstructions were obtained using a global mask to define the overall architecture. Subsequently, local masks were used to perform focused refinements (and occasionally classifications) of individual regions, including the Mlc center and peripheral domains, and bound interaction partners (i.e., DNA, IIB^{Glc} or MtfA). The IIC^{Glc} transporter was separately processed due to high flexibility mediated by the linker between the IIC^{Glc} and IIB^{Glc} proteins (the latter bound to Mlc). Transparent volumes represent lower contour levels of the consensus map. The resulting locally refined and sharpened maps were combined into composite maps that capture the highest local resolution for each region while preserving the native organization of the intact complex. The consensus reconstructions were employed as structural references for placing and aligning the locally refined regions (box). The obtained composite maps were used for model building and structural interpretation. The specific workflows are indicated for (**a**) apo-Mlc, (**b**) the Mlc:DNA complex, (**c**) the Mlc:$IICB^{Glc}$ complex and (**d**) the Mlc:MtfA complex.

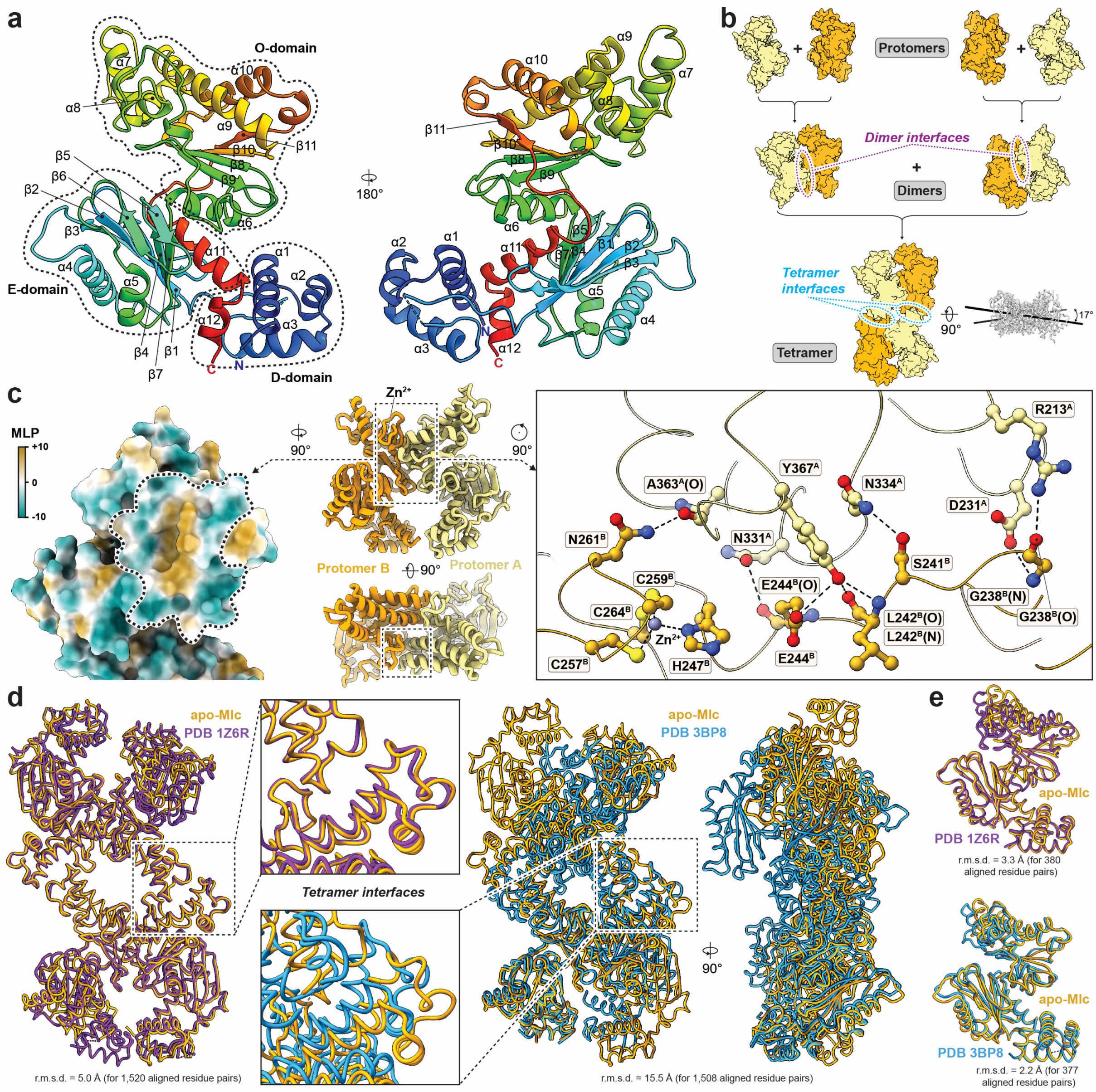

Supplementary Fig. 3: Mlc structure and oligomerization interfaces, and comparison with reported crystal structures. (**a**) Rainbow colored assignment of secondary structure elements in the Mlc protomer. The blue N and red C mark the amino- and carboxy-termini. (**b**) Assembly of the dimer-of-dimers oligomeric Mlc structure, and dimer and tetramer interfaces. (**c**) Orthogonal views (*middle*) of the Mlc dimer interface formed by aligned protomers (orange and yellow). The separated interface (*left*) with dimer interface highlighted (dashed line) colored by hydrophobicity (molecular lipophilicity potential, MLP) reveals a central hydrophobic patch (goldenrod) surrounded by a hydrophilic fringe (dark cyan). Numerous hydrogen bonds (dashed lines) between amino acids contribute to the dimeric interaction between protomers (*right*). N and O in brackets indicate amine and carbonyl backbone interactions, and superscript letters denote the protomers. Additionally, the nearby C3H1-type zinc (Zn^{2+}) finger motif of protomer B is displayed. (**d**) Overall structural alignment of symmetry-related crystallographic tetramers with the apo-Mlc tetramer in solution from cryo-EM. The tetramer observed in 3BP8 appears highly kinked, likely due to crystal packing effects, and therefore does not represent the biologically relevant oligomer conformation and native interface (*inset*). In contrast, the crystallographic tetrameric assembly in PDB 1Z6R and the corresponding tetramer interface are comparable to the cryo-EM Mlc structures, although that study considered the tetramer artificial and reported only the dimeric structure. (**e**) Structural alignments of best fitting promoters of apo-Mlc from cryo-EM with reported X-ray crystallography structures of Mlc (PDBs 1Z6R and 3BP8). Cα root-mean-square deviation (r.m.s.d.) values and numbers of aligned residue pairs are indicated. The bound IIB^{Glc} in PDB 3BP8 is omitted for clarity. PDB, protein data bank identifier.

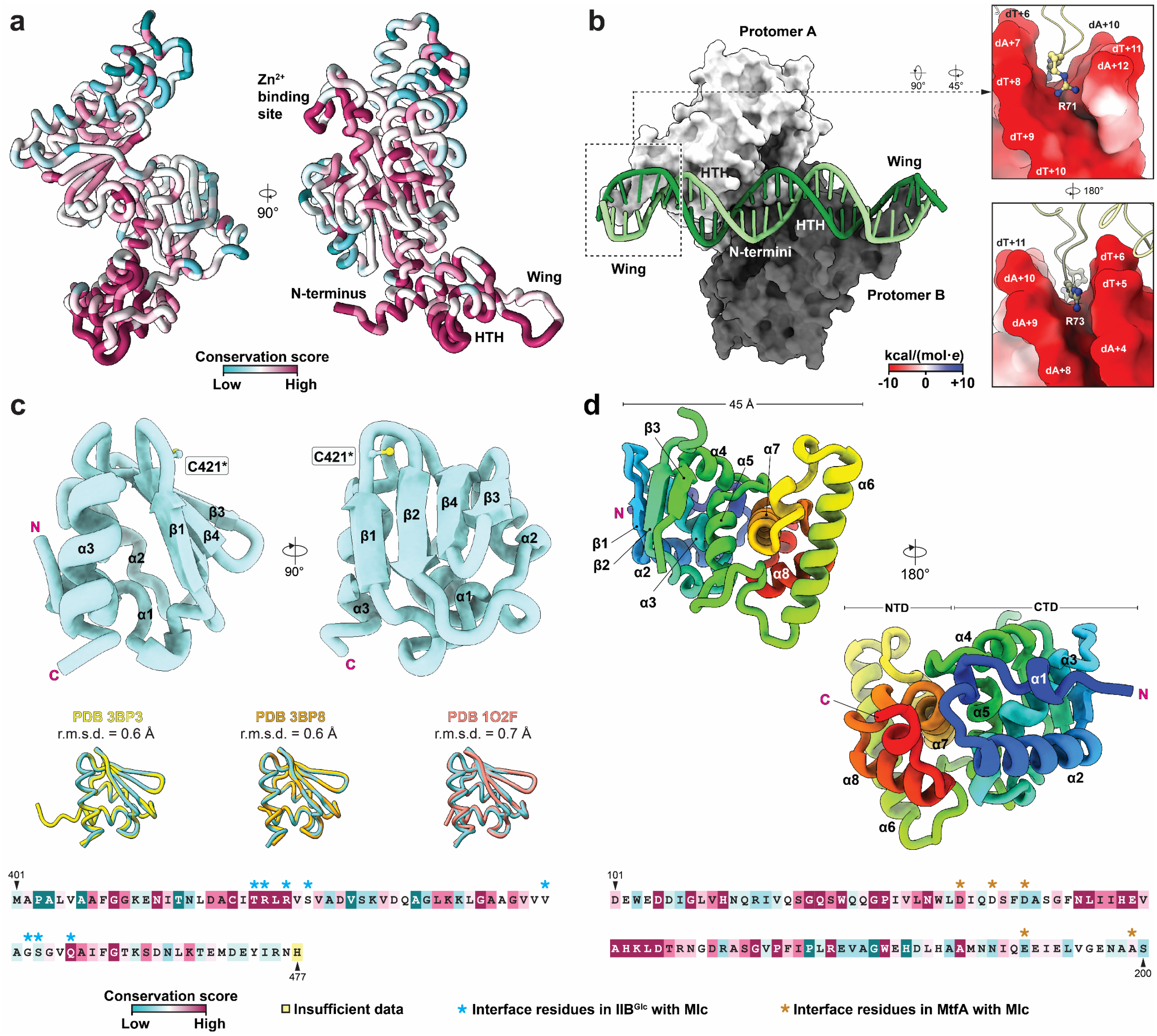

Supplementary Fig. 4: Structures of Mlc complexes and their interaction partners. (**a**) Conservation analysis of Mlc homologues, shown as an Mlc protomer colored according to per-residue conservation scores calculated using the ConSurf algorithm. Variable amino acid residues are shown in turquoise and conserved residues in maroon. The color bar indicates the computed conservation score. (**b**) View of the Mlc dimer (gray tones) bound to DNA (green tones) (*left*) and zoomed-in views of the minor groove (*right*) illustrating arginine residues (R71 and R73) deeply inserted into the DNA minor groove (colored by electrostatic surface potential, color bar in kcal/(mol·*e*)). Backbone phosphates of selected nucleotides are labeled as a guide. (**c**) Structure of IIB^{Glc} in the Mlc:IICB^{Glc} complex with secondary structure assignment (*top*). The active site cysteine (C421) is highlighted, and the letters N and C (pink) mark the amino- and carboxy-termini, respectively. *Bottom*, structural alignment of IIB^{Glc} (in the Mlc:IICB^{Glc} structure) with previously reported IIB^{Glc} crystal structures. The Cα root-mean-square deviations (r.m.s.d.) are indicated. (**d**) Structure of the MtfA monomer in the Mlc:MtfA complex, sequentially rainbow-colored from N- (blue) to C-terminus (red). Dimensions, termini, and subdivision into N- and C-terminal domains (NTD/CTD) are indicated. The per-residue conservation scores calculated using ConSurf are provided for (c) IIB^{Glc} and (d) MtfA, and interfacial residues with Mlc are labeled above with an asterisk (cyan and orange, respectively).

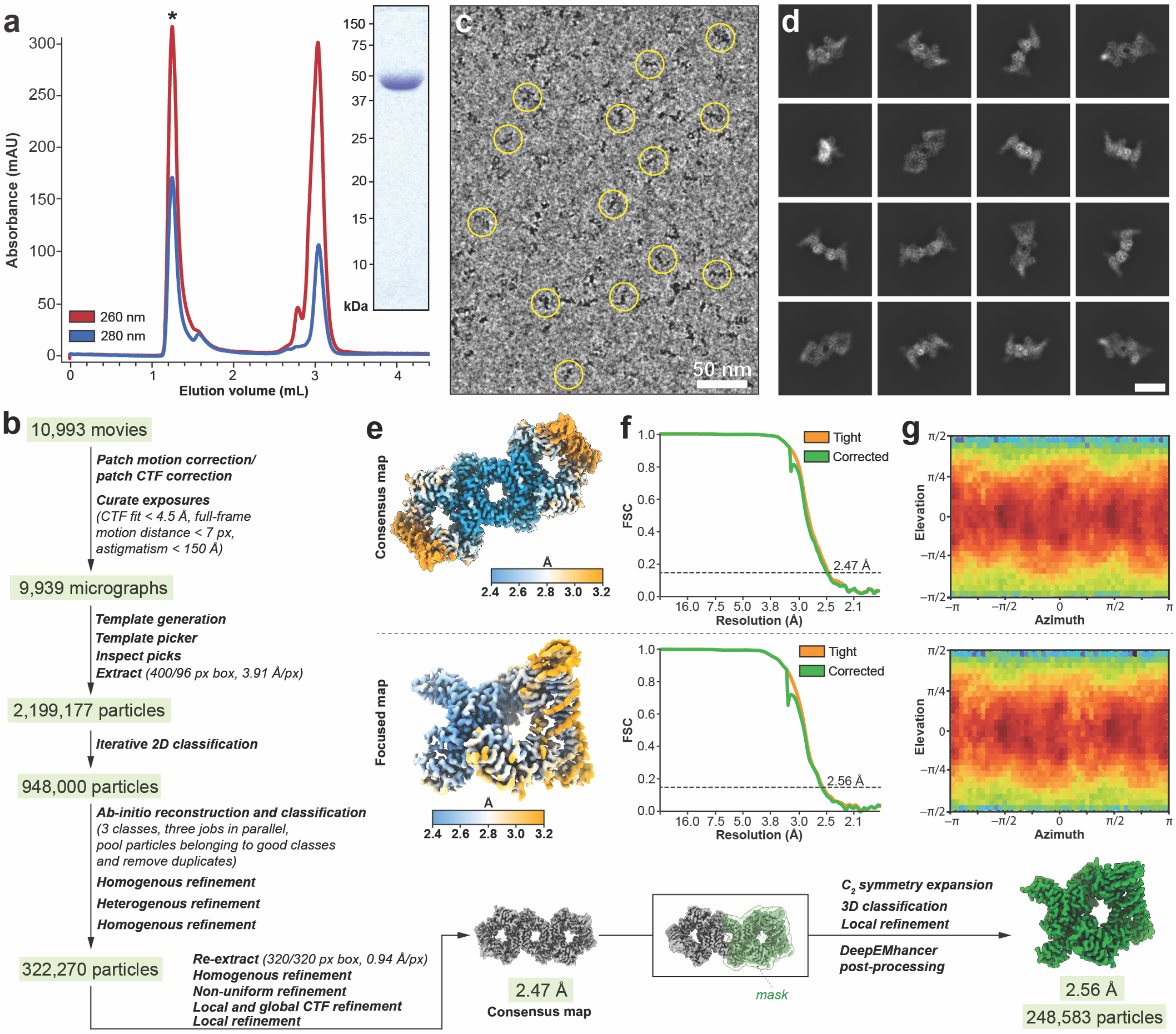

Supplementary Fig. 5: Biochemical characterization, and cryo-EM single-particle analysis, 3D reconstruction and validation of Mlc in complex with the *ptsG* operon ($ptsG^{op}$) (Mlc:DNA). (**a**) Analytical size-exclusion chromatogram of the assembled Mlc:DNA complex. The peak at ~1.2 mL corresponds to the Mlc:DNA complex (marked with an asterisk) and the peak at ~3 mL corresponds to excess DNA. Absorbances at 260 (red) and 280 nm (blue) are indicated. SDS-PAGE analysis (*inset*) of the Mlc:DNA complex shows Mlc (~44 kDa); the DNA fragment (~18 kDa) is not stained by Coomassie Brilliant Blue. Source data are provided as a Source Data file. (**b**) Data processing workflow. The soft mask (transparent green surface) used for focused classification and local refinement is indicated (box). Further details on data processing are provided in the methods section. (**c**) Exemplary electron micrograph (at 1.8 µm defocus, low pass filtered) with representative particles highlighted by yellow circles. (**d**) Representative 2D class averages. The scale bar represents 10 nm. (**e**) Cryo-EM density maps: consensus and locally refined reconstructions colored by computed local resolutions. Color bars indicate resolution in Å. (**f**) Fourier-shell correlation (FSC) plots. (**g**) Viewing-direction distribution plot showing the number of particles at specific elevation and azimuth angles contributing to the final reconstructions. Red means more and blue fewer particles of the specific viewing directions.

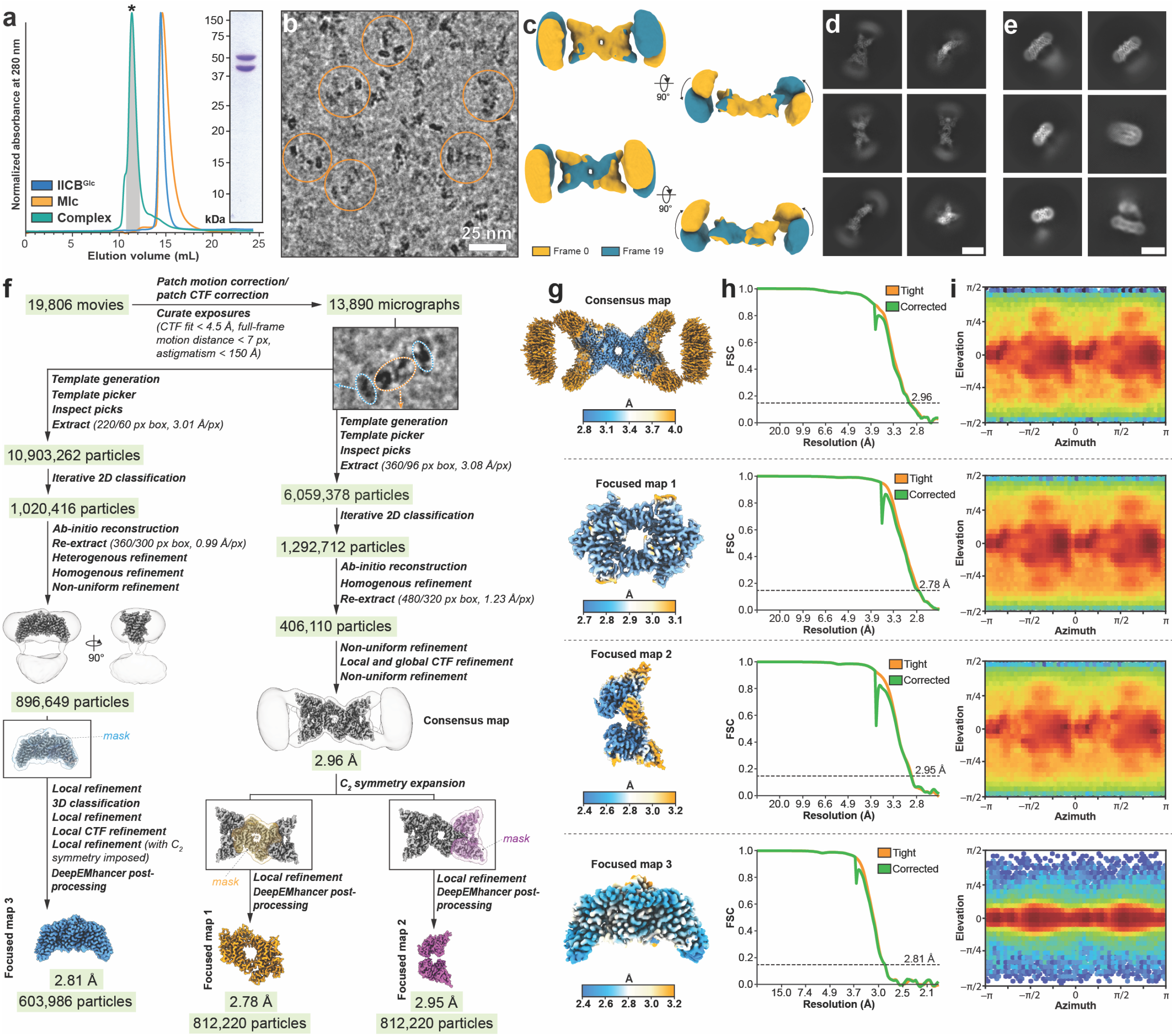

Supplementary Fig. 6: Biochemical characterization, and cryo-EM single-particle analysis, 3D reconstruction and validation of MIc in complex with the glucose transporter IICB^{Glc} (MIc:IICB^{Glc}). (**a**) Size-exclusion chromatography analysis of the MIc:IICB^{Glc} complex, and MIc and IICB^{Glc} run individually. The fraction used for cryo-EM is shaded in gray. The inset shows a Coomassie Brilliant Blue-stained SDS-PAGE gel of the complex peak fraction (asterisk), revealing equimolar amounts of MIc (~44 kDa) and IICB^{Glc} (~52 kDa). Source data are provided as a Source Data file. (**b**) Exemplary electron micrograph (at 2.1 µm defocus, low pass filtered) with representative particles highlighted by orange circles. (**c**) Cryo-EM 3D variability analysis (3DVA) of MIc:IICB^{Glc} revealing a continuous rock (*top*) and scissor (*bottom*) movement of peripheral densities as principal modes of variability, prompting an individual data processing strategy. (**d**) and (**e**) display representative 2D class averages from the processing of the central and peripheral parts of the complex, respectively. Scale bars represent 10 nm. (**f**) Data processing workflow. The central (MIc and IIB^{Glc}, orange ellipse) and peripheral (IIC^{Glc}, blue ellipses) parts of the complex were processed separately. The soft masks (transparent surfaces) used for focused classification and local refinement are indicated (boxes). Further details on data processing are provided in the methods section. (**g**) Cryo-EM density maps: consensus and locally refined colored by computed local resolutions. Color bars indicate resolution in Å. (**h**) Fourier-shell correlation (FSC) plots. (**i**) Viewing-direction distribution plots showing the number of particles at specific elevation and azimuth angles contributing to the final reconstruction. Red means more and blue fewer particles of the specific viewing directions.

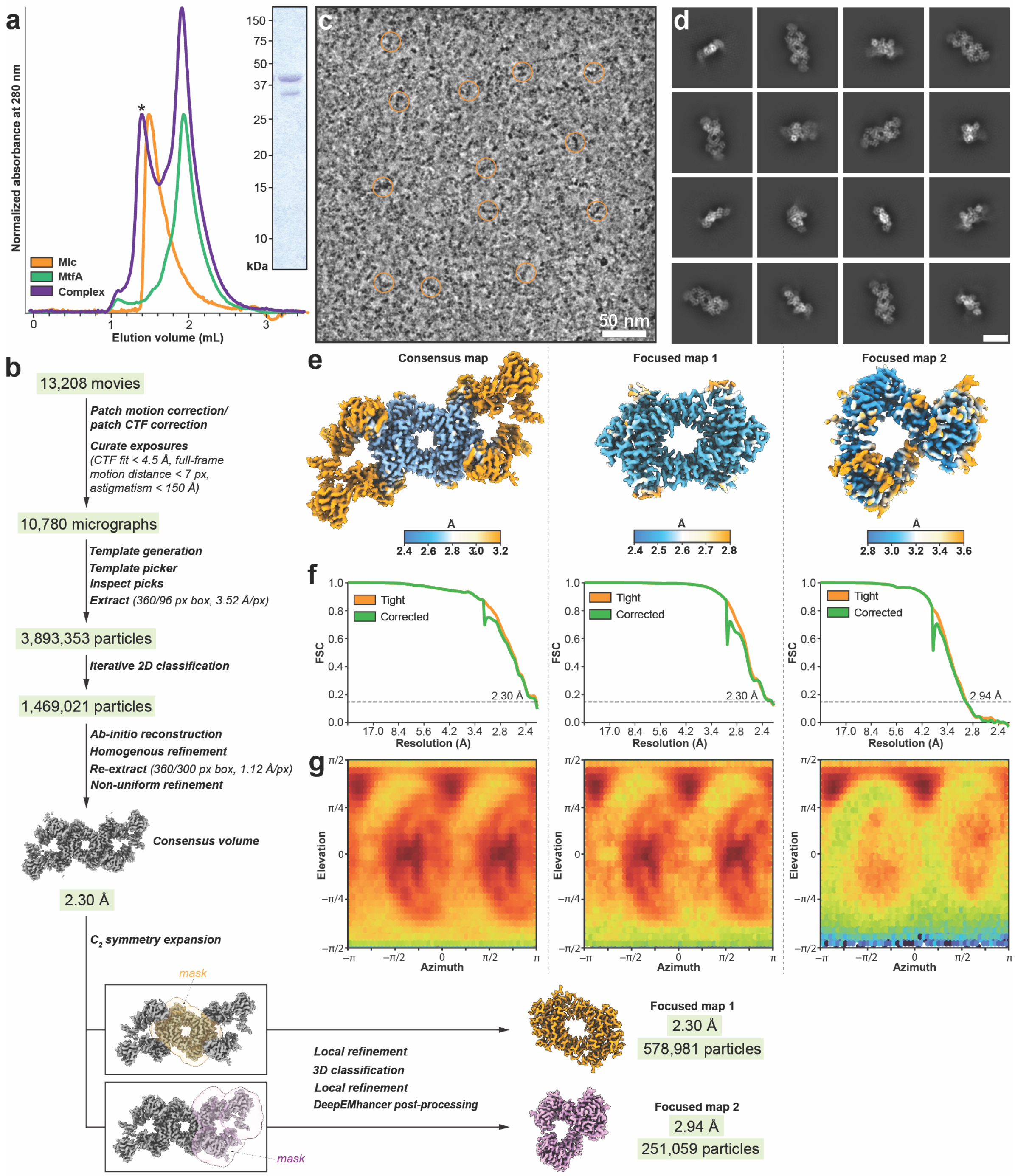

Supplementary Fig. 7: Biochemical characterization, and cryo-EM single-particle analysis, 3D reconstruction and validation of Mlc in complex with the anti-repressor MtfA (Mlc:MtfA). (**a**) Analytical size-exclusion chromatography (SEC) analysis of the Mlc:MtfA complex, and Mlc and MtfA run individually (normalized to the complex peak for comparison, marked with an asterisk). Coomassie Brilliant Blue-stained SDS-PAGE gel (*inset*) of the complex peak fraction reveals co-elution of Mlc (~44 kDa) and MtfA (~30 kDa). Source data are provided as a Source Data file. (**b**) Data processing workflow. The soft masks (transparent surfaces) used for focused classification and local refinement are indicated (boxes). Further details on data processing are provided in the methods section. (**c**) Exemplary electron micrograph (at 1.9 µm defocus, low pass filtered) with representative particles highlighted by orange circles. (**d**) Representative 2D class averages. The scale bar represents 10 nm. (**e**) Cryo-EM density maps: Consensus and locally refined maps are colored by computed local resolutions. Color bars indicate the resolution in Å. (**f**) Fourier-shell correlation (FSC) plots. (**g**) Viewing-direction distribution plot showing the number of particles at specific elevation and azimuth angles contributing to the final reconstruction. Red means more and blue fewer particles of the specific viewing directions.

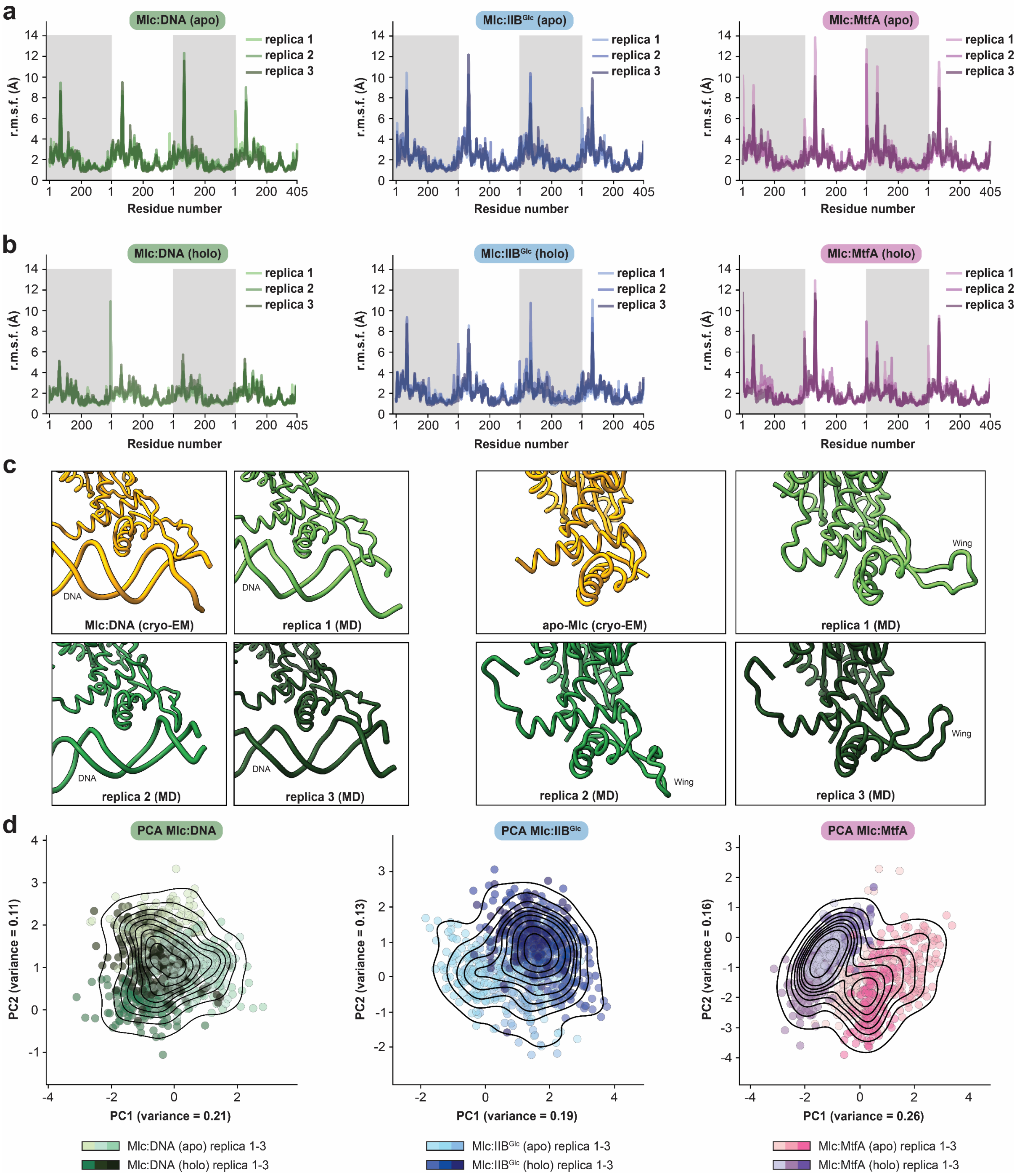

Supplementary Fig. 8: Comparative dynamics of Mlc complexes assessed by molecular dynamics (MD) simulations. Cα root-mean-square fluctuation (r.m.s.f.) per residue plots for (**a**) apo and (**b**) holo trajectories, i.e., for Mlc:DNA, Mlc:IIB^{Glc} and Mlc:MtfA. Gray and white boxes denote the four Mlc protomers. (**c**) Structural comparison between cryo-EM models and representative MD simulation snapshots (centroid structures from the distance analysis in Fig. 5d). Operator interactions are shown for the Mlc:DNA cryo-EM structure and corresponding snapshots from three independent Mlc:DNA (holo) simulations (*left*), and for the apo-Mlc cryo-EM structure with representative snapshots from three independent Mlc:DNA (apo) simulations (*right*), which closely match the experimental models. The wing revealed high flexibility in the apo-Mlc cryo-EM structure, in line with the flexibility observed in the apo simulation replicas. (**d**) Principal component analysis (PCA) plots of apo (light colors) and holo (dark colors) complexes sorted by interaction partner. Data points are provided for each of the three replicas.

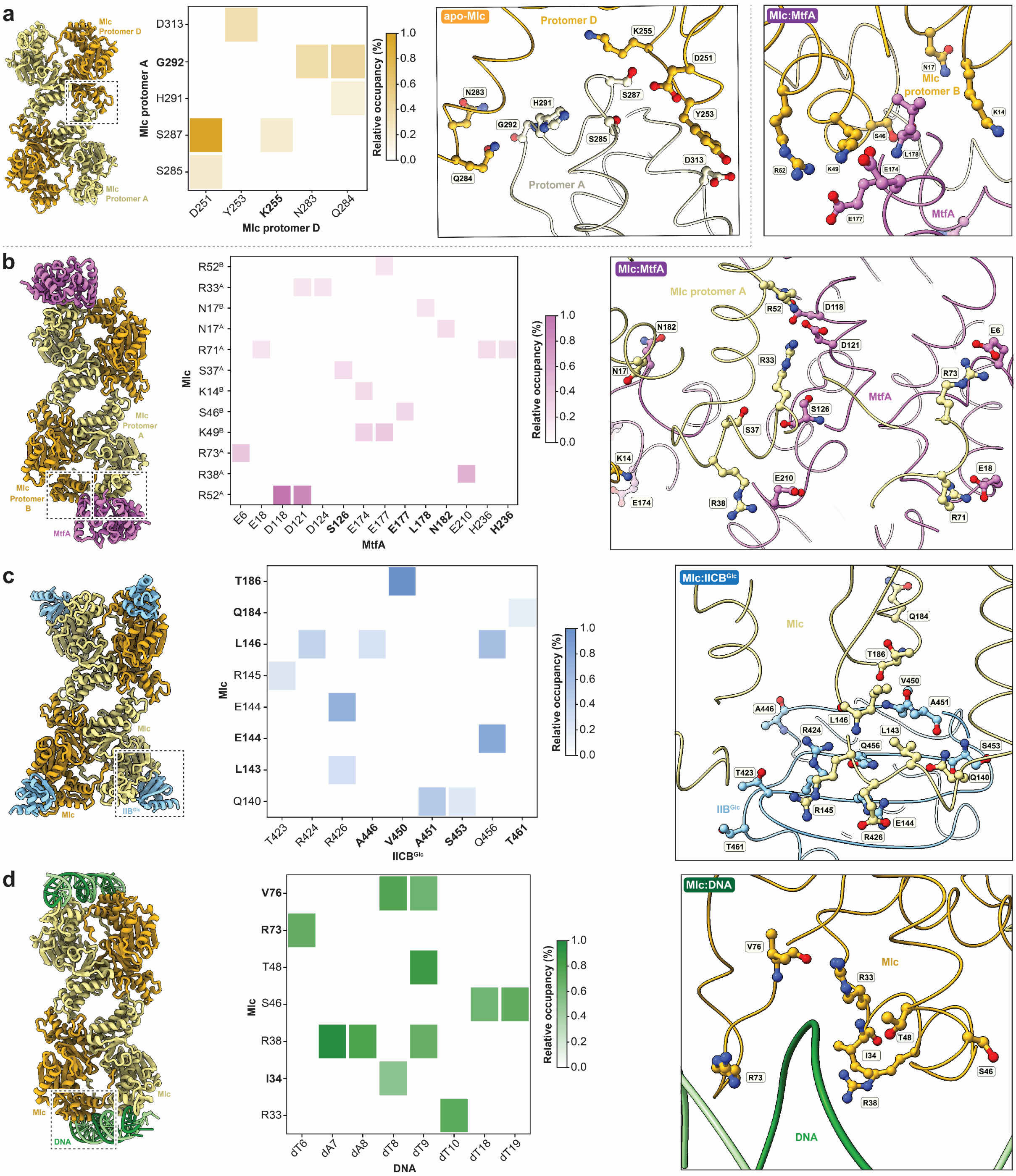

Supplementary Fig. 9: Dynamics of interfacial residues analyzed by molecular dynamics (MD) simulations. For each interaction, i.e., (**a**) the Mlc tetramer interface (top 10% of interactions shown), (**b**) Mlc:MtfA, (**c**) Mlc:IICB^{Glc} and (**d**) Mlc:DNA, an overview of the interaction interface, a heatmap of the relative frequency of the top 5% interactions and an illustration of the identified high-frequency contacts (amino acid-amino acid or amino acid-nucleotide) are presented. Interacting Mlc protomers are colored in orange and yellow, MtfA in purple, IIB^{Glc} in sky blue and DNA strands in green. Amino acid residues in bold represent backbone interactions, i.e., involving the backbone amine or carbonyl groups. For the Mlc:MtfA interaction, Mlc protomers are indicated with superscript A or B. Nucleotides correspond to the numbers in the PDB file.

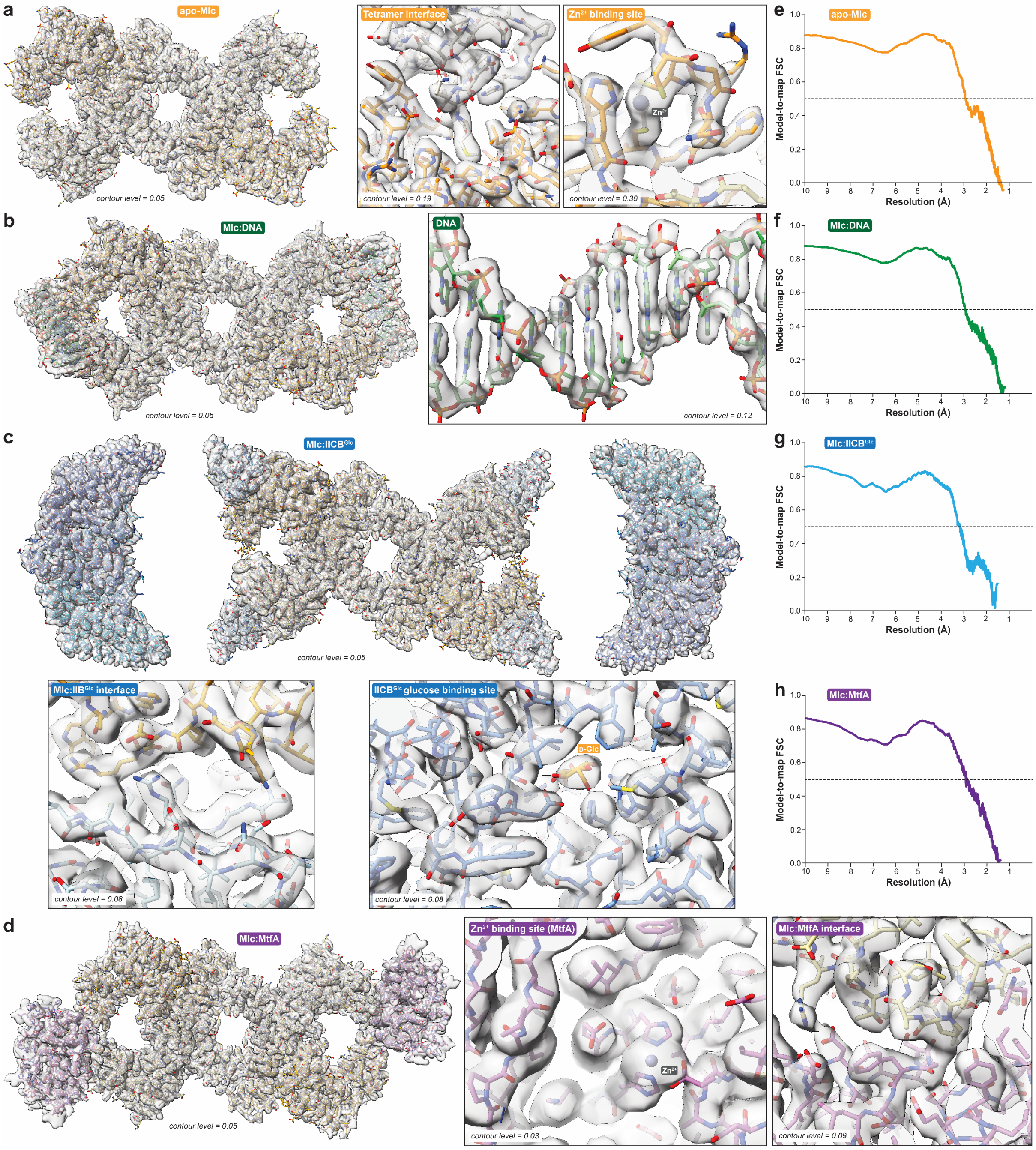

Supplementary Fig. 10: Gallery of representative cryo-EM densities (transparent gray surfaces) with corresponding fitted atomic models (model-to-map fit) for the Mlc complexes reported in this study. The shown densities are derived from the respective composite maps, and the contour levels are indicated (no carving was applied). (**a**) Apo-Mlc in orange/yellow with a zoomed-in view of the tetramer interface and the Zn^{2+} (gray sphere) binding site. (**b**) Mlc:DNA complex (Mlc in orange/yellow, DNA in green) with a close-up view of a DNA segment within the binding region. (**c**) Mlc:IICB^{Glc} complex (Mlc in orange/yellow, IICB^{Glc} in shades of blue) with detailed views of the Mlc:IIB^{Glc} interface and the glucose-binding site within IIC^{Glc}, showing the bound glucose molecule (D-Glc; orange). (**d**) Mlc:MtfA complex (Mlc in orange/yellow, MtfA in purple) and zoomed-in view of the Zn^{2+} (gray sphere) binding site in MtfA. Panels **(e-h)** display the model-to-map Fourier-shell correlation (FSC) plots for the reported Mlc complexes. The FSC = 0.5 criterion is indicated with a dashed line.

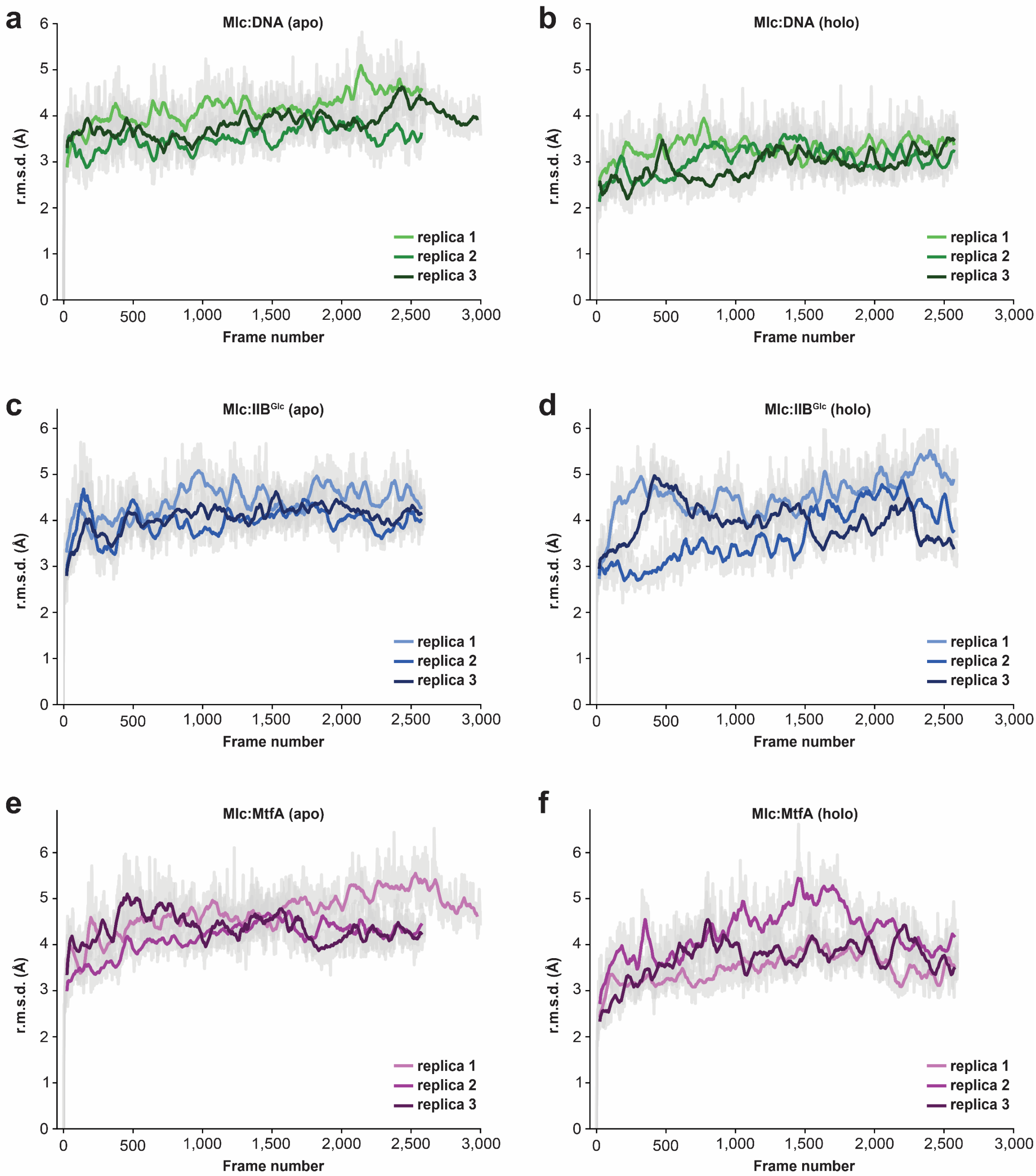

Supplementary Fig. 11: Cα root-mean-square deviation (r.m.s.d.) of Mlc obtained from molecular dynamics simulations. The r.m.s.d. over time is shown for Mlc in complex with its binding partners (holo) and in the corresponding interactor-free states (apo). For each complex, three independent simulations (replicas) are displayed as moving averages (colored lines) and raw data (gray). Colors correspond to Mlc:DNA (green), Mlc:IIB^{Glc} (blue) and Mlc:MtfA (purple). All simulations were initiated from the experimental cryo-EM structures, with missing residues modelled where necessary. Frames were written every 0.5 ns.

Supplementary Table 1: Molecular dynamics simulation setup parameters.

System	Starting structure (PDB ID)	Number of atoms	Box dimensions (Å)	replica	Simulation time (μs)	Number of atoms	Box dimensions (Å)	replica	Simulation time (μs)
		holo				apo			
Mlc:DNA	9SRU	841,633	207 × 209 × 204	1	1.3	700,792	192 × 196 × 196	1	1.3
				2	1.3			2	1.3
				3	1.3			3	1.5
Mlc:IIB^{Glc}	9SRX	765,438	203 × 200 × 197	1	1.3	625,627	181 × 184 × 197	1	1.3
				2	1.3			2	1.3
				3	1.3			3	1.3
Mlc:MtfA	9SRW	813,329	206 × 207 × 200	1	1.3	644,605	196 × 182 × 189	1	1.5
				2	1.3			2	1.3
				3	1.3			3	1.3
