# Supplementary material for: Structural basis of Mlc-mediated transcriptional regulation of carbohydrate metabolism
Source: Nat Commun. 2026 Jul 11;17:8553. doi: 10.1038/s41467-026-75270-8 (PMC13482132; doi:10.1038/s41467-026-75270-8)
Supplement: Supplementary file 2 [file 41467_2026_75270_MOESM2_ESM.pdf]

Description of Additional Supplementary Files

File Name: Supplementary Data 1

Description: Cryo-EM data collection, refinement and validation statistics.
