# Supplementary material for: Structural basis of Mlc-mediated transcriptional regulation of carbohydrate metabolism
Source: Nat Commun. 2026 Jul 11;17:8553. doi: 10.1038/s41467-026-75270-8 (PMC13482132; doi:10.1038/s41467-026-75270-8)
Supplement: Supplementary file 4 [file 41467_2026_75270_MOESM5_ESM.pdf]

nature portfolio

Peer Review File

Structural basis of Mlc-mediated transcriptional regulation of carbohydrate metabolism

Corresponding Author: Professor Dimitrios Fotiadis

This file contains all reviewer reports in order by version, followed by all author rebuttals in order by version.

Version 0:

Reviewer comments:

Reviewer #1

(Remarks to the Author)
The manuscript titled “Structural basis of Mlc-mediated transcriptional regulation of carbohydrate metabolism” by Roth et al. describes the structural studies of Mlc in its apo and holo forms. The study reports the cryo-EM structures of apo Mlc, Mlc bound to its cognate DNA operator sequence, Mlc bound to the glucose-specific PTS transporter IICB, and anti-repressor MtfA. Overall, the results of this study are novel and enhance the understanding of gene regulation in E. coli at the protein-protein interaction level. Although most of the structures are well resolved, the structure of the Mlc-IICB complex is not very convincing. For example, the gap between the Mlc-IIB and IIC protomers is large. It is not very clear how this gap is filled in the physiological condition. It has not been discussed in detail.

Reviewer #2

(Remarks to the Author)
The manuscript "Structural basis of Mlc-mediated transcriptional regulation of carbohydrate metabolism," presents a series of cryo-EM structures characterizing the E. coli Mlc repressor.
The research is technically robust, providing a high-resolution structural atlas of Mlc in four functional states: the apo-tetramer, a DNA-bound complex, a membrane-transporter bound complex (IICBGlc), and an anti-repressor bound complex (MtfA). The most noteworthy result is the discovery that Mlc functions as a rigid homotetrameric scaffold. This challenges the common assumption that such repressors require large-scale allosteric conformational changes to function. Instead, the data demonstrates that Mlc’s activity is regulated through steric exclusion and direct protein-protein competition. The structural evidence for non-canonical DNA recognition—where disordered "wings" (specifically residues R71 and R73) become ordered to bind the minor groove - is well-supported by the maps and provides a clear mechanism for operator specificity.

Significance and Comparison to Literature
This work is significant because it resolves long-standing questions regarding the oligomeric state of Mlc, confirming it as a dimer-of-dimers tetramer in solution rather than an artifact of crystallization. It provides the first high-resolution framework for the sequestration of a transcription factor by a membrane-embedded transporter (IICBGlc), a key aspect of the phosphotransferase system (PTS). The methodology meets the highest standards in the field, utilizing state-of-the-art cryo-EM data collection and 23.8 µs of molecular dynamics (MD) simulations to bridge the gap between static structures and biological dynamics. The methods are described with sufficient detail to allow for reproduction, and the reporting summaries are transparent.
Points for Revision and Improvement
While the structural data is excellent, a few areas require clarification or minor additions before publication:
• Local Resolution in the IICB Complex: The Mlc: (IICBGlc), complex shows lower local resolution in the membrane-proximal regions. The authors should explicitly discuss whether this reflects inherent biological plasticity or sample heterogeneity.
• Integration of MD Data: The manuscript would benefit from a more direct link between the MD simulations and the structural snapshots. Specifically, it should be clarified if the simulations show the peripheral DNA-binding domains sampling orientations seen in the bound states.
• Functional Validation: Although the structural interfaces are convincing, adding in vivo reporter assays (such as LacZ assays) with mutations at the "wing" residues (R71/R73) or the MtfA binding interface would provide necessary biological validation of the cryo-EM models.
• Zinc Finger Role: The study identifies a C3H1-type zinc finger. A brief discussion or supplemental data addressing

whether this motif is purely structural for tetramer stability or plays a role in environmental sensing would strengthen the discussion.
Finally, the manuscript is a comprehensive study that provides a clear structural basis for Mlc regulation. Provided the authors address the minor points regarding local resolution and functional validation of the binding interfaces, the work is suitable for publication.

Reviewer #3

(Remarks to the Author)

Summary

The manuscript by Roth et al. investigates the regulation of Mlc, a transcriptional repressor that controls genes involved in bacterial glucose metabolism. It is thought that protein-protein interactions mediate Mlc regulation, but this is poorly understood at a molecular level. To better understand this principle of regulation, the authors determine four single-particle cryo-electron microscopy (cryo-EM) structures of Mlc (apo), Mlc bound to a cognate DNA operator, Mlc in complex with phosphoenolpyruvate (PEP)-dependent phosphotransferase system (PTS) glucose transporter $IICB^{Glc}$, and Mlc in complex with MtfA. These high-resolution structures were then coupled with molecular dynamics simulations to uncover the dynamics of Mlc in the presence of these regulatory factors. This study builds on our understanding of how transcription factors are regulated by environmental effectors (e.g., small molecules) by establishing how factors such as Mlc may be regulated through protein-protein interactions.

The manuscript is well-written, and the structural data are sound and clearly presented. I recommend this manuscript for acceptance into Nature Communications if the following critiques are addressed:

Major Comments

(1) In their structures, authors observe that Mlc adopts a dimer-of-dimers homo-tetrameric assembly and that DNA, $IICB^{Glc}$, and MtfA bind the dimers on both sides of the complex. This is likely due to high protein concentrations during sample preparation and a molar excess of factor relative to the Mlc dimer, and therefore represents a limitation in the in vitro reconstitution of these complexes. Can tetrameric Mlc bind multiple DNA sites in the genome? If so, what is the function for this?

In their discussion, the author should comment on the number of Mlc DNA-binding sites versus the amounts of Mlc, $IICB^{Glc}$, and MtfA in the cell, and whether more complex arrangements can form and their implications for regulatory interplay (e.g., Mlc homo-tetramers bound to DNA on one side and $IICB^{Glc}$ on the other side). Could their molecular dynamics (MD) simulations inform us on the conformational dynamics of Mlc in these more complicated arrangements?

(2) Based on their structures, authors propose two models for how Mlc repression is sequestered: (i) Mlc binding to the IIB^{Glc} domain of the $IICB^{Glc}$ transporter leads to localization to the plasma membrane, and (ii) binding of MtfA onto the D-domain of Mlc sterically excludes DNA. It is hard to imagine that Mlc localization to the plasma membrane alone is sufficient to sequester Mlc DNA binding, primarily since the IIB^{Glc} domain mainly binds the Mlc E-domain, and the DNA-binding interface of Mlc is still accessible. Could these models be additive? The additive model would entail titration of MtfA, leading to binding of both D-domains and outcompeting binding to DNA operators. The open E-domains bind to $IICB^{Glc}$ and localize Mlc to the membrane. Based on the experimental structures and MD, the binding of MftA should not exclude IIB^{Glc}.

Could the authors please elaborate on this in their discussion and revise Fig. 6 (currently shows structural comparisons) to a model figure for how regulation occurs? This model should summarize their structural findings and the potential interplay of these different factors. This will help provide a framework for the impact of their findings and motivation for future studies.

(3) In their data processing of single-particle cryo-EM datasets, the authors combine separate refinement maps into a composite map, often from different particle pools or subsets. In each sample, the author should confirm that the overall conformation of the complex remains unchanged across the different particle pools.

An example of this is seen in the processing of apo-Mlc (Extended Data Fig. 1). The authors use 3D classification followed by local refinement to improve resolution in the D- and E-domains of Mlc. These locally refined maps were then combined with the consensus to generate a composite map. However, the use of 3D classification creates a discrepancy between the particles that contribute to the consensus map and those that contribute to the locally refined map. 679,852 particles from the consensus refinement were used to generate focused map 1, which was then stitched with focused map 2, which only comes from a subset of these particles (244,307 particles). Is the overall conformation of the entire complex the same in this subset of particles compared to the conformation observed in the map comprising the 679,852 particles?

In the Mlc:$IICB^{Glc}$ sample, the $IICB^{Glc}$ (603,986 particles) and Mlc tetramer (406,110 particles) were picked separately, yet the resultant maps were combined into a single composite map. Do the $IICB^{Glc}$ picked particles have full occupancy for Mlc tetramer (and vice versa)? Are they in similar confirmations to one another?

On a related note, some parts of the authors' cryoEM pipelines lack key details (e.g., the number of rounds of 3D classification, how masks were created, and how composite maps were stitched). The authors should include sufficient detail so that others may reproduce their processing scheme.

Minor Comments
(1) To help readers better understand the quality of the cryoEM maps and their fitted models, authors should include examples of map-model fit by highlighting different parts of their models with corresponding densities, noting the sigma level and carve distance. Authors should also include map-model FSCs for each sample.

(2) The linker between $IICB^{Glc}$ transporter and the IIB^{Glc} domain is poorly resolved in the Mlc:$IICB^{Glc}$ map. Could the authors use 3D variability analysis (as in the apo-Mlc dataset) to examine the conformational heterogeneity of these linkers?

(3) Extended Data Fig. 3a – The authors should disclose more information about which sequences and the number of sequences used for conservation analysis. Related to this, how conserved are the residues in $IICB^{Glc}$ and MtfA that make up the interfaces with Mlc?

(4) Extended Data Fig. 3b – Please include labels for amino acid residues and DNA base pairs.

(5) Extended Data Fig. 6a – Purple peak is cut off in the chromatogram.

Version 1:

Reviewer comments:

Reviewer #1

(Remarks to the Author)
The questions raised by this reviewer have been taken care of by the authors in the revised version of the manuscript.

Reviewer #2

(Remarks to the Author)
In the manuscript, Roth et al. investigate the structural mechanisms behind how the global transcriptional repressor Mlc regulates carbohydrate metabolism in Escherichia coli. Using cryo-EM and molecular dynamics simulations, the authors present high-resolution structures of Mlc in its apo state, as well as in complex with its cognate operator DNA, the PTS transporter IICB-Glc, and the anti-repressor MtfA. Together, these distinct functional states provide a clear, comprehensive model for how Mlc switches between DNA binding, membrane sequestration, and cytosolic regulation. Showing the atomic interfaces between a soluble repressor and a membrane-embedded transporter provides valuable insights for both structural biology and microbial physiology.

The authors have successfully addressed the feedback from the previous round of review. Broadly, the changes were made to clarify specific structural measurements, improve the description of the protein-protein and protein-DNA interfaces, and refine the discussion. The validation reports confirm that the structural models fit the experimental density maps very well, and the updated text ensures the proposed competitive binding mechanisms are thoroughly supported.

The cryo-EM data collection, processing, and refinement workflows follow standard, high-quality practices in the field, and the reporting summary contains all necessary details for reproducibility. Because the revisions have fully resolved the previous issues and the manuscript is now clear and technically sound, I recommend accepting the paper for publication.

Reviewer #3

(Remarks to the Author)
The authors have made substantial improvements to the manuscript and have addressed the majority of my previous concerns. In particular, they have improved the clarity of the text, expanded the discussion of Mlc assembly and regulation, and improved transparency in cryo-EM data processing. Overall, I believe the manuscript is suitable for publication in Nature Communications.

Open Access This Peer Review File is licensed under a Creative Commons Attribution 4.0 International License, which permits use, sharing, adaptation, distribution and reproduction in any medium or format, as long as you give appropriate credit to the original author(s) and the source, provide a link to the Creative Commons license, and indicate if changes were made.
In cases where reviewers are anonymous, credit should be given to 'Anonymous Referee' and the source.
The images or other third party material in this Peer Review File are included in the article's Creative Commons license, unless indicated otherwise in a credit line to the material. If material is not included in the article's Creative Commons license and your intended use is not permitted by statutory regulation or exceeds the permitted use, you will need to obtain permission directly from the copyright holder.
To view a copy of this license, visit https://creativecommons.org/licenses/by/4.0/

Point-by-point response for Roth et al. (NCOMMS-25-96518-T)

Authors: We thank the three Reviewers for their careful evaluation of our manuscript and for providing insightful and constructive comments. We appreciate the opportunity to clarify and expand on our work. All suggestions have been considered and the manuscript has been revised accordingly. In addition, we have refined the text throughout to improve clarity. Changes and additions are highlighted in the tracked changes version of the revised manuscript (provided as a PDF alongside the clean version) and detailed point-by-point responses to each comment are given below. As a note, Extended and Supplementary Figures have been merged into a single set of Supplementary Figures, in line with the presentation style of *Nature Communications*.

Reviewer #1

The manuscript titled "Structural basis of Mlc-mediated transcriptional regulation of carbohydrate metabolism" by Roth et al. describes the structural studies of Mlc in its apo and holo forms. The study reports the cryo-EM structures of apo Mlc, Mlc bound to its cognate DNA operator sequence, Mlc bound to the glucose-specific PTS transporter IICB, and anti-repressor MtfA. Overall, the results of this study are novel and enhance the understanding of gene regulation in E. coli at the protein-protein interaction level. Although most of the structures are well resolved, the structure of the Mlc-IICB complex is not very convincing. For example, the gap between the Mlc-IIB and IIC protomers is large. It is not very clear how this gap is filled in the physiological condition. It has not been discussed in detail.

Authors : We thank the Reviewer for their positive assessment of this work. From a technical perspective, and in response to comments by Reviewers #2 and #3, we have added more details on data processing and the generation of the presented composite structures. In particular, the new Supplementary Figure 2 illustrates how the composite maps were derived from focused and consensus maps. We hope this offers clearer insight into, for example, the structure determination of the Mlc-IICB complex.

Furthermore, we have addressed the Reviewer's comment on the apparent separation between Mlc:IIB^{Glc} and IIC^{Glc} in the presented structure: see the last sentence of the second paragraph of Discussion.

Reviewer #2

The manuscript "Structural basis of Mlc-mediated transcriptional regulation of carbohydrate metabolism," presents a series of cryo-EM structures characterizing the E. coli Mlc repressor. The research is technically robust, providing a high-resolution structural atlas of Mlc in four functional states: the apo-tetramer, a DNA-bound complex, a membrane-transporter bound complex (IICBGlc), and an anti-repressor bound complex (MtfA). The most noteworthy result is the discovery that Mlc functions as a rigid homotetrameric scaffold. This challenges the common assumption that such repressors require large-scale allosteric conformational changes to function. Instead, the data demonstrates that Mlc's activity is regulated through steric exclusion and direct protein-protein competition. The structural evidence for non-canonical DNA recognition—where disordered "wings" (specifically residues R71 and R73)

become ordered to bind the minor groove - is well-supported by the maps and provides a clear mechanism for operator specificity.

Significance and Comparison to Literature
This work is significant because it resolves long-standing questions regarding the oligomeric state of Mlc, confirming it as a dimer-of-dimers tetramer in solution rather than an artifact of crystallization. It provides the first high-resolution framework for the sequestration of a transcription factor by a membrane-embedded transporter (IICBGlc), a key aspect of the phosphotransferase system (PTS). The methodology meets the highest standards in the field, utilizing state-of-the-art cryo-EM data collection and 23.8 μs of molecular dynamics (MD) simulations to bridge the gap between static structures and biological dynamics. The methods are described with sufficient detail to allow for reproduction, and the reporting summaries are transparent.

Authors: We thank the Reviewer for their constructive and positive evaluation.

Points for Revision and Improvement
While the structural data is excellent, a few areas require clarification or minor additions before publication:
• Local Resolution in the IICB Complex: The Mlc: (IICBGlc), complex shows lower local resolution in the membrane-proximal regions. The authors should explicitly discuss whether this reflects inherent biological plasticity or sample heterogeneity.

Authors: We thank the Reviewer for raising this point. The lower local resolution (Extended Data Fig. 5g [original submission] and Supplementary Fig. 6g [revised manuscript]) is observed in the consensus map of the whole Mlc:IICB^{Glc} complex, i.e., prior to focused refinements. While the central Mlc:IIB^{Glc} part is well resolved, the relative position of the IIC^{Glc} membrane transporters appears less well defined, as shown in the 3DVA results and 2D class averages (Extended Data Fig. 5c [original submission] and Supplementary Fig. 6c-e [revised manuscript]). This particle heterogeneity is likely due to the intrinsically flexible linker between the IIB^{Glc} and IIC^{Glc} domains, as noted earlier for the isolated IICB^{Glc} complex (PMIDs: 39266522 and 40124667). In the revised manuscript, we address this point: see the last sentence of the second paragraph of Discussion. In addition, we provide the new Supplementary Fig. 2 to further clarify the generation of the presented composite maps.

• Integration of MD Data: The manuscript would benefit from a more direct link between the MD simulations and the structural snapshots. Specifically, it should be clarified if the simulations show the peripheral DNA-binding domains sampling orientations seen in the bound states.

Authors: The authors thank the Reviewer for this helpful suggestion to strengthen the link between the MD simulations and the experimentally observed structural snapshots. To address this point, we have added an additional panel in Supplementary Figure 8c that illustrates the consistency between the cryo-EM data and the MD simulations. Specifically and as requested, we compare cryo-EM models with representative MD snapshots of apo-Mlc and Mlc:DNA, highlighting the flexibility of the D-domain wings in the apo state and their more ordered interactions upon binding to the DNA operator.

• Functional Validation: Although the structural interfaces are convincing, adding in vivo reporter assays (such as LacZ assays) with mutations at the "wing" residues (R71/R73) or the MtfA binding interface would provide necessary biological validation of the cryo-EM models.

Authors: We thank the Reviewer for this suggestion and agree that functional validation of the identified interfaces would corroborate the structural findings. However, mutational analyses targeting the Mlc "wing" residues as well as the MtfA interaction interface have already been reported in previous studies and provide already strong *in vivo* and *in vitro* support for their functional relevance (PMIDs: 22788997 and 25452338 [Mlc wing arginine resdues], and PMID: 22178967 [MtfA interaction interface]). In light of this existing body of evidence, we have not repeated such experiments here. Instead, in the revised manuscript, we now explicitly discuss, acknowledge and reference these studies in the Discussion to better contextualize and support our structural and MD-based findings: see the second half of the second paragraph of the Discussion.

• Zinc Finger Role: The study identifies a C3H1-type zinc finger. A brief discussion or supplemental data addressing whether this motif is purely structural for tetramer stability or plays a role in environmental sensing would strengthen the discussion.

Authors: We thank the Reviewer for highlighting this point. ROK family members with known structures, including repressors and related kinases, typically feature a zinc binding motif (C3H1 or C2H2) defining the ROK fold. While conceivable, no environmental sensing role has been reported for ROK family members. Based on available structures, the C3H1-type zinc finger in Mlc suggests a structural role, likely contributing to the stability and proper folding of the assembly. Supporting this, Schiefner et al. (2005) (PMID: 15929984) proposed a structural role for the zinc ion in Mlc based on mutagenesis. In the revised manuscript, we have extended Supplementary Fig. 3c to show the zinc finger near the dimer interface in Mlc (now described in the Results section), highlighting its potential role in stabilizing the oligomeric assembly, and have added a corresponding statement in the Discussion section: see second paragraph, third sentence.

Finally, the manuscript is a comprehensive study that provides a clear structural basis for Mlc regulation. Provided the authors address the minor points regarding local resolution and functional validation of the binding interfaces, the work is suitable for publication.

Authors: We thank the Reviewer for the positive feedback and constructive comments, which have helped improve the manuscript and have been addressed in the revised manuscript.

Reviewer #3

Summary

The manuscript by Roth et al. investigates the regulation of Mlc, a transcriptional repressor that controls genes involved in bacterial glucose metabolism. It is thought that protein-protein interactions mediate Mlc regulation, but this is poorly understood at a molecular level. To better understand this principle of regulation, the authors determine four single-particle cryo-electron microscopy (cryo-EM) structures of Mlc (apo), Mlc bound to a cognate DNA operator, Mlc in complex with phosphoenolpyruvate (PEP)-dependent phosphotransferase system (PTS) glucose transporter $IICB^{Glc}$, and Mlc in complex with MtfA. These high-resolution structures were then coupled with molecular dynamics simulations to uncover the dynamics of Mlc in the presence of these regulatory factors. This study builds on our understanding of how transcription factors are regulated by environmental effectors (e.g., small molecules) by establishing how factors such as Mlc may be regulated through protein-protein interactions.

The manuscript is well-written, and the structural data are sound and clearly presented. I recommend this manuscript for acceptance into Nature Communications if the following critiques are addressed:

Authors: We appreciate this Reviewer's thorough review and the positive assessment of our work.

Major Comments

(1) In their structures, authors observe that Mlc adopts a dimer-of-dimers homo-tetrameric assembly and that DNA, IICBGlc, and MtfA bind the dimers on both sides of the complex. This is likely due to high protein concentrations during sample preparation and a molar excess of factor relative to the Mlc dimer, and therefore represents a limitation in the in vitro reconstitution of these complexes. Can tetrameric Mlc bind multiple DNA sites in the genome? If so, what is the function for this?

Authors: To obtain homogeneous samples for cryo-EM, the complexes were assembled with binding partners in excess relative to Mlc to ensure saturation of the binding sites, enabling structurally well-defined results. Multiple operator sites targeted by Mlc have been reported, sharing partially conserved pseudo-palindromic DNA sequence architectures (Fig. 2a, bottom portion of the panel). The presence of two operator-binding sites in tetrameric Mlc could, in principle, enable simultaneous binding to multiple operators. However, this possibility cannot be directly addressed with the current experimental approaches. In summary, such higher-order assemblies are conceivable, and we have added a corresponding statement in the Discussion: see third paragraph, last two sentences.

In their discussion, the author should comment on the number of Mlc DNA-binding sites versus the amounts of Mlc, $IICB^{Glc}$, and MtfA in the cell, and whether more complex arrangements can form and their implications for regulatory interplay (e.g., Mlc homo-tetramers bound to DNA on one side and $IICB^{Glc}$ on the other side). Could their molecular dynamics (MD) simulations inform us on the conformational dynamics of Mlc in these more complicated arrangements?

Authors: We thank the Reviewer for this valuable comment. Our structural data on four key Mlc complexes indeed raise the possibility that hetero-complexes may form in vivo, e.g. with one Mlc dimer bound to DNA and the other engaging a protein interaction partner, potentially adding an additional layer of regulatory control. At present, however, quantitative data on the relative and absolute cellular levels of Mlc, $IICB^{Glc}$ and MtfA are scarce and not systematically characterized across physiological conditions, although their interplay is likely to be functionally relevant. We have therefore included a statement discussing this potential regulatory interplay in the revised manuscript (Discussion: third paragraph, last two sentences).

We agree that molecular dynamics (MD) simulations of such higher-order assemblies could provide further insights into the conformational dynamics of Mlc in these more complex arrangements. However, modeling these systems would require substantially larger assemblies and extended sampling times beyond the scope of the present study. The MD simulations presented here were specifically designed to probe the intrinsic flexibility of Mlc within the experimentally resolved complexes. Exploration of larger, higher-order assemblies will be a possible direction for future work.

(2) Based on their structures, authors propose two models for how Mlc repression is sequestered: (i) Mlc binding to the IIB^{Glc} domain of the $IICB^{Glc}$ transporter leads to localization to the plasma membrane, and (ii) binding of MtfA onto the D-domain of Mlc sterically excludes DNA. It is hard to imagine that Mlc localization to the plasma membrane alone is sufficient to sequester Mlc DNA binding, primarily since the IIB^{Glc} domain mainly binds the Mlc E-domain, and the DNA-binding interface of Mlc is still accessible. Could these models be additive? The additive model would entail titration of MtfA, leading to binding of both D-domains and outcompeting binding to DNA operators. The open E-domains bind to $IICB^{Glc}$ and localize Mlc to the membrane. Based on the experimental structures and MD, the binding of MftA should not exclude IIB^{Glc}.

Authors: We thank the Reviewer for this thoughtful and mechanistically well-reasoned comment. Both $IICB^{Glc}$-mediated membrane sequestration and MtfA-mediated inhibition have been reported in the literature to independently relieve Mlc repression. We agree that membrane localization alone may not fully account for the loss of DNA binding, particularly given that the IIB^{Glc} domain engages the E-domain of Mlc, while leaving the DNA-binding interface accessible. In this context, our structural data are compatible with a potential functional interplay between these mechanisms. For example, the spatial separation of the IIB^{Glc} and MtfA binding sites suggests that simultaneous binding of both partners is sterically feasible, consistent with the Reviewer's proposal. However, whether these mechanisms act additively or cooperatively within the same complex or instead represent distinct regulatory inputs remains unclear. Given the lack of direct experimental evidence and the anticipated complexity of the system, we consider a more detailed mechanistic interpretation to be beyond the scope of the present study.

Could the authors please elaborate on this in their discussion and revise Fig. 6 (currently shows structural comparisons) to a model figure for how regulation occurs? This model should summarize their structural findings and the potential interplay of these different factors. This will help provide a framework for the impact of their findings and motivation for future studies.

Authors: We thank the Reviewer for this suggestion. In the revised manuscript, we have added a schematic panel to Fig. 6 that complements the structural comparison and summarizes characterized mechanisms of Mlc regulation based on the literature. In line with our previous response, we present these regulatory aspects cautiously, focusing on experimentally supported mechanisms and avoiding speculation on potential additive or cooperative effects. Nevertheless, we believe that this panel provides a useful conceptual overview of the system and helps to contextualize our findings while motivating future studies on Mlc regulation.

(3) In their data processing of single-particle cryo-EM datasets, the authors combine separate refinement maps into a composite map, often from different particle pools or subsets. In each sample, the author should confirm that the overall conformation of the complex remains unchanged across the different particle pools.

An example of this is seen in the processing of apo-Mlc (Extended Data Fig. 1). The authors use 3D classification followed by local refinement to improve resolution in the D- and E-domains of Mlc. These locally refined maps were then combined with the consensus to generate a composite map. However, the use of 3D classification creates a discrepancy between the particles that contribute to the consensus map and those that contribute to the locally refined map. 679,852 particles from the consensus refinement were used to generate

focused map 1, which was then stitched with focused map 2, which only comes from a subset of these particles (244,307 particles). Is the overall conformation of the entire complex the same in this subset of particles compared to the conformation observed in the map comprising the 679,852 particles?
In the Mlc:IICB^{Glc} sample, the IICB^{Glc} (603,986 particles) and Mlc tetramer (406,110 particles) were picked separately, yet the resultant maps were combined into a single composite map. Do the IICB^{Glc} picked particles have full occupancy for Mlc tetramer (and vice versa)? Are they in similar confirmations to one another?

On a related note, some parts of the authors' cryoEM pipelines lack key details (e.g., the number of rounds of 3D classification, how masks were created, and how composite maps were stitched). The authors should include sufficient detail so that others may reproduce their processing scheme.

Authors: We acknowledge this methodological shortcoming and have updated the revised manuscript with further details on cryo-EM data processing and interpretation. Particularly, we have extended the Methods section and the Supplementary Figures to include additional details on the cryo-EM pipelines, including the number of rounds of 3D classification, the strategy used for mask generation and the procedures used for focused refinement. In addition, given the central importance of composite map generation for this work and for transparency, we have included the new Supplementary Fig. 2 that details this procedure. Furthermore, we now provide detailed explanations in the Methods section on how the composite maps were obtained. The overlays of consensus and aligned focused maps (Supplementary Fig. 2, third row) demonstrate the consistency with the generated composite maps. Therefore, we can confirm that the use of particle subsets after further particle classifications and focused refinements did not impact the overall conformation.

In the Mlc:IICB^{Glc} complexes, full occupancy of both Mlc and IICB^{Glc} (and vice versa) is supported by their consistent presence in the micrographs used for analysis. Furthermore, the 3DVA results and the 2D class averages corroborate this observation (see Supplementary Fig. 6b-e).

Minor Comments
(1) To help readers better understand the quality of the cryoEM maps and their fitted models, authors should include examples of map-model fit by highlighting different parts of their models with corresponding densities, noting the sigma level and carve distance. Authors should also include map-model FSCs for each sample.

Authors: We thank the Reviewer for this suggestion. Examples illustrating map to model fit of the structures are now provided in Suppl. Fig. 10. As requested, we have added the corresponding contour levels and noted that no carving was applied. In addition, model-to-map FSC curves (as calculated in Phenix) are now also included for each structure.

(2) The linker between IICBGlc transporter and the IIBGlc domain is poorly resolved in the Mlc:IICBGlc map. Could the authors use 3D variability analysis (as in the apo-Mlc dataset) to examine the conformational heterogeneity of these linkers?

Authors: We thank the Reviewer for this suggestion. We did consider applying 3D variability analysis (3DVA) to probe conformational heterogeneity of the linker between the IIC^{Glc} transporter and the IIB^{Glc} domain. However, meaningful 3DVA requires a consistently resolvable density that can be isolated by masking. In the Mlc:IICB^{Glc} dataset, the linker region

is not visible in the consensus map, consistent with its high flexibility, as also reported for the isolated IICB^{Glc} complex (PMIDs: 39266522 and 40124667). This observation suggests pronounced flexibility rather than discrete conformational states. As a result, it is not possible to define a mask encompassing this linker and exploratory 3DVA runs did not yield interpretable variability modes. We therefore conclude that the linker behaves as a highly flexible tether that does not adopt stable conformations detectable by cryo-EM and we have clarified this point in the revised manuscript (last sentence of the second paragraph of Discussion).

(3) Extended Data Fig. 3a – The authors should disclose more information about which sequences and the number of sequences used for conservation analysis. Related to this, how conserved are the residues in IICB^{Glc} and MtfA that make up the interfaces with Mlc?

Authors: The sequence conservation analyses calculated using the ConSurf algorithm (default parameters) are now described in the Methods section (under *Miscellaneous*). As requested, we further include the conservation analyses for IIB^{Glc} and MtfA residues interacting with Mlc at the interfaces (see Supplementary Figure 4c (IIB^{Glc}) and d (MtfA), bottom row).

(4) Extended Data Fig. 3b – Please include labels for amino acid residues and DNA base pairs.

Authors: We labelled the requested residues and bases in Suppl. Fig. 4b (previously Extended Data Fig. 3b).

(5) Extended Data Fig. 6a – Purple peak is cut off in the chromatogram.

Authors: This panel was adapted accordingly, see Suppl. Fig. 7a (previously Extended Data Fig. 6a).

Point-by-point response for Roth et al. (NCOMMS-25-96518-A)

Reviewer #1

The questions raised by this reviewer have been taken care of by the authors in the revised version of the manuscript.

Authors: We thank the reviewer for their positive assessment.

Reviewer #2

In the manuscript, Roth et al. investigate the structural mechanisms behind how the global transcriptional repressor Mlc regulates carbohydrate metabolism in Escherichia coli. Using cryo-EM and molecular dynamics simulations, the authors present high-resolution structures of Mlc in its apo state, as well as in complex with its cognate operator DNA, the PTS transporter IICB-Glc, and the anti-repressor MtfA. Together, these distinct functional states provide a clear, comprehensive model for how Mlc switches between DNA binding, membrane sequestration, and cytosolic regulation. Showing the atomic interfaces between a soluble repressor and a membrane-embedded transporter provides valuable insights for both structural biology and microbial physiology.

The authors have successfully addressed the feedback from the previous round of review. Broadly, the changes were made to clarify specific structural measurements, improve the description of the protein-protein and protein-DNA interfaces, and refine the discussion. The validation reports confirm that the structural models fit the experimental density maps very well, and the updated text ensures the proposed competitive binding mechanisms are thoroughly supported.

The cryo-EM data collection, processing, and refinement workflows follow standard, high-quality practices in the field, and the reporting summary contains all necessary details for reproducibility. Because the revisions have fully resolved the previous issues and the manuscript is now clear and technically sound, I recommend accepting the paper for publication.

Authors: We are grateful for the reviewer's constructive feedback during the review process and thank the reviewer for recommending acceptance.

Reviewer #3

The authors have made substantial improvements to the manuscript and have addressed the majority of my previous concerns. In particular, they have improved the clarity of the text, expanded the discussion of Mlc assembly and regulation, and improved transparency in cryo-EM data processing. Overall, I believe the manuscript is suitable for publication in Nature Communications.

Authors: We thank the reviewer for the positive assessment of the revised manuscript and for supporting publication.
